# MiT/TFE Family of Transcription Factors: An Evolutionary Perspective

**DOI:** 10.3389/fcell.2020.609683

**Published:** 2021-01-06

**Authors:** Martina La Spina, Pablo S. Contreras, Alberto Rissone, Naresh K. Meena, Eutteum Jeong, José A. Martina

**Affiliations:** Cell and Developmental Biology Center, National Heart, Lung, and Blood Institute, National Institutes of Health, Bethesda, MD, United States

**Keywords:** lysosomes, autophagy, mammalian target of rapamycin (mTOR), transcription factor EB (TFEB), transcription factor E3 (TFE3), helix-loop-helix transcription factor 30 (HLH-30), microphthalmia-associated transcription factor (MITF), evolution

## Abstract

Response and adaptation to stress are critical for the survival of all living organisms. The regulation of the transcriptional machinery is an important aspect of these complex processes. The members of the microphthalmia (MiT/TFE) family of transcription factors, apart from their involvement in melanocyte biology, are emerging as key players in a wide range of cellular functions in response to a plethora of internal and external stresses. The MiT/TFE proteins are structurally related and conserved through evolution. Their tissue expression and activities are highly regulated by alternative splicing, promoter usage, and posttranslational modifications. Here, we summarize the functions of MiT/TFE proteins as master transcriptional regulators across evolution and discuss the contribution of animal models to our understanding of the various roles of these transcription factors. We also highlight the importance of deciphering transcriptional regulatory mechanisms in the quest for potential therapeutic targets for human diseases, such as lysosomal storage disorders, neurodegeneration, and cancer.

## Graphical Abstract

MiT/TFE transcription factors are master regulators of cellular adaptation to a wide variety of stressful conditions. They control the expression of a plethora of genes involved in response to nutrient deprivation, oxidative and ER stress, and DNA and mitochondrial damage. MiT/TFE proteins play a critical role in organelle biogenesis, control of energy homeostasis, adaptation to pathogen infection, control of growth and development, aging, and death. MiT/TFE proteins are also modulators of critical signaling pathways that regulate cell proliferation, cellular fate commitment, and tumorigenesis. Many of these functions are evolutionary conserved from lower metazoans to mammals indicating that the adaptation to challenging conditions occurred early during evolution.

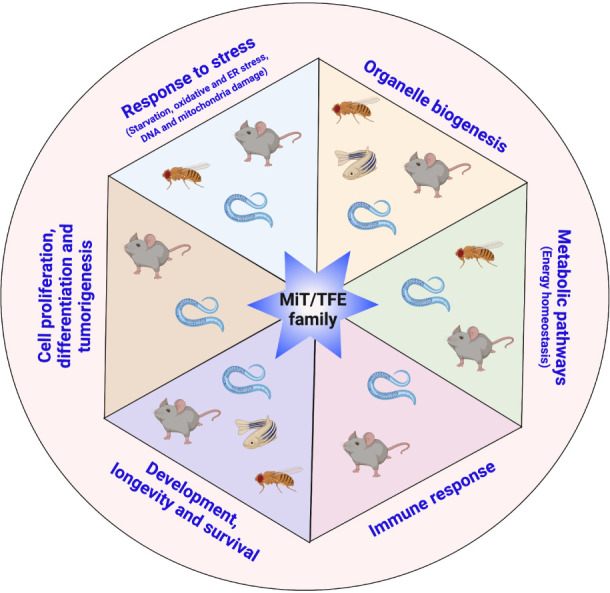

## Introduction

The microphthalmia (MiT/TFE) transcription factors belong to the superfamily of functionally unrelated basic helix-loop-helix leucine zipper (bHLH-ZIP)-containing proteins that includes transcription regulators, such as Myc, Max, sterol regulatory element-binding protein (SREBP), Mad, upstream stimulatory factor (USF), MAX-Like Factor X (MLX), and activating enhancing binding protein 4 (AP4) (Ledent and Vervoort, 2001). In vertebrates, the MiT/TFE family is composed of four evolutionarily conserved and closely related members: microphthalmia-associated transcription factor (MITF), transcription factor EB (TFEB), TFE3, and TFEC (Steingrimsson et al., 2004). These transcription factors can form homodimers and heterodimers and bind, through the basic domain, to the regulatory regions of their target genes that contain a palindromic 6-base pair CANNTG motif, termed E-box (Hemesath et al., 1994; Pogenberg et al., 2020). Analysis of the promoters of many lysosomal genes revealed the presence of a palindromic 10-base pair motif (GTCACGTGAC), a type of E-box, called CLEAR (coordinated lysosomal expression and regulation) element (Sardiello et al., 2009). Both TFEB and TFE3 were shown to directly bind to the CLEAR motif (Palmieri et al., 2011; Martina et al., 2014b).

MiT/TFE proteins are key players in many fundamental cellular processes, and their regulation is essential for organismal adaptation to challenges imposed by a wide variety of both internal and external cues (Puertollano et al., 2018). In addition to the role of MiT/TFE proteins as master regulators of lysosomal function and autophagy, there is a wealth of evidence demonstrating their involvement in an ever-expanding list of cellular processes including nutrient sensing, energy metabolism, response to endoplasmic reticulum (ER) stress, mitochondrial and DNA damage, oxidative stress, innate immunity and inflammation, longevity, cellular survival, cell fate decisions, neurodegeneration, and cancer (Raben and Puertollano, 2016; Slade and Pulinilkunnil, 2017; Cortes and La Spada, 2019; Dall and Faergeman, 2019; Goding and Arnheiter, 2019; Bahrami et al., 2020; Irazoqui, 2020; Wang S. et al., 2020).

**FIGURE 1:**
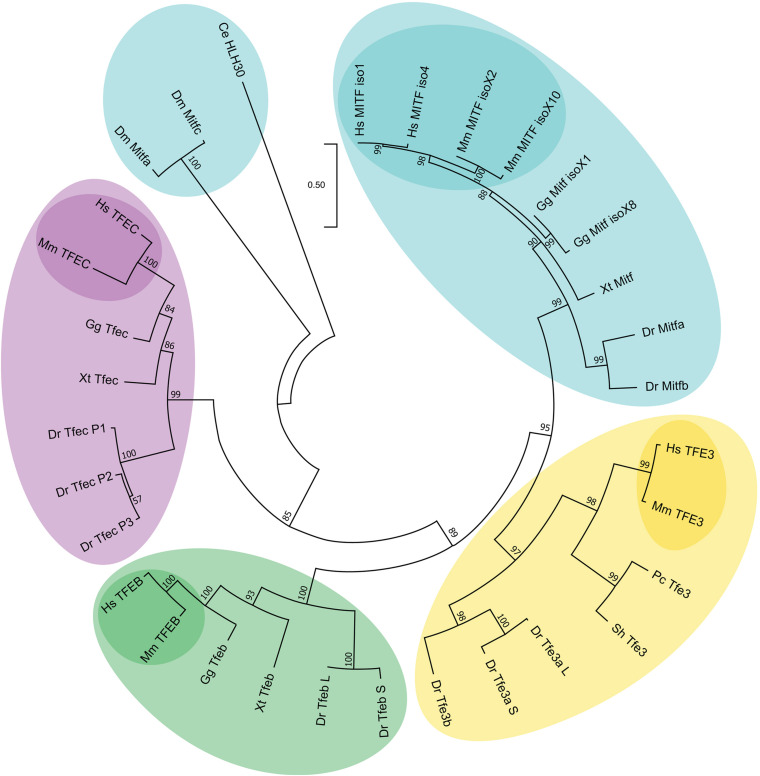
Phylogenetic relationships of the different MiT/TFE family members. Evolutionary comparison of different members of the MiT/TFE protein family represented in a phylogenetic rooted tree generated using MEGA X program (ver. 10.1.8) with a Maximum Likelihood and JTT matrix-based model and 1,000 bootstrap replicates (Jones et al., 1992; Kumar et al., 2018). The tree with the highest log likelihood (–17751.05) is shown. The percentage of trees in which the associated taxa clustered together is shown next to each branch. The tree is drawn to scale, with branch lengths measured in the number of substitutions per site. The scale of the branch lengths is indicated below the tree. The four MiT/TFE members are highlighted in different colors. Mammalian proteins are highlighted in darker colors. Ce, *Caenorhabditis elegans*; Dm, *Drosophila melanogaster*; Dr, *Danio rerio*; Gg, *Gallus gallus*; Hs, *Homo sapiens*; L, long; Mm, *Mus musculus*; P1-3, protein 1–3; Pc, *Phasianus colchicus*; S, short; Sh, *Strigops habroptila*; Xt, *Xenopus tropicalis*.

MiT/TFE proteins are conserved through evolution, and their homologs can be found in primitive metazoans, such as *Trichoplax* and sponges (Simionato et al., 2007; Gyoja, 2014). Phylogenetic analysis clearly shows that vertebrate MiT/TFE proteins are sorted in four clades, each representing one of the orthologs of the family, whereas the invertebrate orthologs, helix-loop-helix transcription factor 30 (HLH-30) in *Caenorhabditis elegans* (Rehli et al., 1999; Lapierre et al., 2013) and Mitf in *Drosophila melanogaster* (Hallsson et al., 2004), are out-grouped (Figure 1 and Supplementary Table 1). The four mammalian MiT/TFE family members most likely originated from a common ancestor gene that underwent two rounds of whole-genome duplications (WGDs); an additional round of WGD is believed to occur in the zebrafish genome that contains a total of six genes encoding for MiT/TFE proteins (Mitfa, Mitfb, Tfe3a, Tfe3b, Tfeb, and Tfec) (Taylor et al., 2001; Lister et al., 2011). The ability of a single MiT/TFE transcription factor in invertebrates to perform specific (basal) functions that are carried out by different specialized orthologs in vertebrates strongly argues for a functional differentiation process of these proteins during evolution (Zhang et al., 2015; Kuo et al., 2018; Dall and Faergeman, 2019; Goding and Arnheiter, 2019).

Early studies indicated that the six major human MITF isoforms originated from a combination of multiple alternative splicing events and promoter usage, resulting in the expression of MITF proteins with different amino termini (Goding and Arnheiter, 2019). MITF-M is the melanocyte-specific isoform mostly associated with the regulation of pigmentation, melanocyte development and differentiation, deafness, and melanoma biology. Interestingly, MITF-M can induce the expression of a subset of lysosomal and autophagy genes, suggesting a possible role of this isoform in the regulation of lysosomal biogenesis and autophagy in melanoma (Ploper et al., 2015; Leclerc et al., 2019; Moller et al., 2019). Moreover, the activity/nucleus–cytoplasm shuttling of the ubiquitously expressed non-melanocyte MITF-A isoform is regulated by a mechanism that controls the expression of several autophagy genes and involves Rag GTPases, 14-3-3, and mammalian target of rapamycin complex 1 (mTORC1) proteins (Martina and Puertollano, 2013; Martina et al., 2014a). These MITF functions have been recently comprehensively reviewed (Kawakami and Fisher, 2017; Goding and Arnheiter, 2019) and will not be discussed further.

**FIGURE 2:**
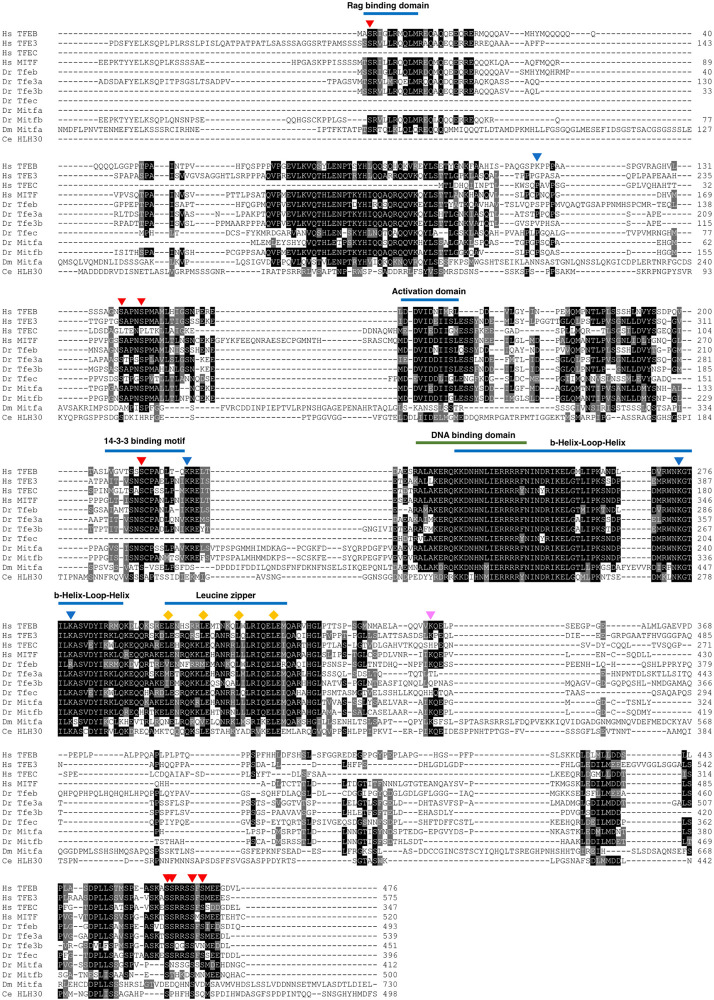
Sequence conservation of MiT/TFE transcription factors across species. Clustal Omega multiple sequence alignment of MiT/TFE proteins from *Homo sapiens* (Hs), *Danio rerio* (Dr), *Drosophila melanogaster* (Dm), and *Caenorhabditis elegans* (Ce). Shaded boxes highlight the degree of conservation of key functional domains between all proteins and species. Arrowheads indicate posttranslational modifications described in human TFEB, showing different degrees of conservation between species. 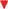 Phosphorylation at serines 3, 138, 142, 211, 462, 463, 467, and 469; 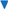 Acetylation at lysines 116, 219, 274, and 279; 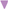 Sumoylation at lysine 346. Diamonds (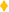) point out leucine residues 298, 305, 312, and 319 important for the leucine zipper domain function. Note that the N-terminal sequences of some of the proteins analyzed were omitted due to the figure size constraints.

All members of the MiT/TFE family share highly conserved functional domains across different species, including those required for DNA binding and homo/heterodimerization (Figure 2). Significant similarity is also observed within their N- and C-terminal regions that play a major role in the regulation of protein localization and stability. Moreover, MiT/TFE proteins are subjected to a variety of posttranslational modifications such as phosphorylation, acetylation, SUMOylation, oxidation, and ubiquitination (Puertollano et al., 2018; Goding and Arnheiter, 2019; Wang et al., 2019). Of note, some key residues are conserved across species, suggesting that the basal regulation of the MiT/TFE factors is evolutionarily conserved (Figure 2) (Settembre et al., 2011; Martina et al., 2012; Roczniak-Ferguson et al., 2012; Settembre et al., 2012; Chang et al., 2018).

In this review, we summarize our current knowledge of these transcription factors from an evolutionary perspective, mainly focusing on stress response and adaptation. We discuss how studies in different animal models could provide insights into the regulatory mechanisms governing cellular functions and facilitate the development of new therapeutic strategies to combat a range of human diseases.

## Regulation of MiT/TFE Activation in Response to Nutrient Deprivation

Living organisms are continuously challenged by a variety of stresses. Eukaryotic cells rely on the tightly controlled defense pathways to cope with adverse conditions, thus enabling proper growth and aging, while dysregulation of these pathways may have fatal consequences. The members of the MiT/TFE family of transcription factors play pivotal roles in the maintenance of cellular homeostasis in response to a variety of stress conditions.

**FIGURE 3:**
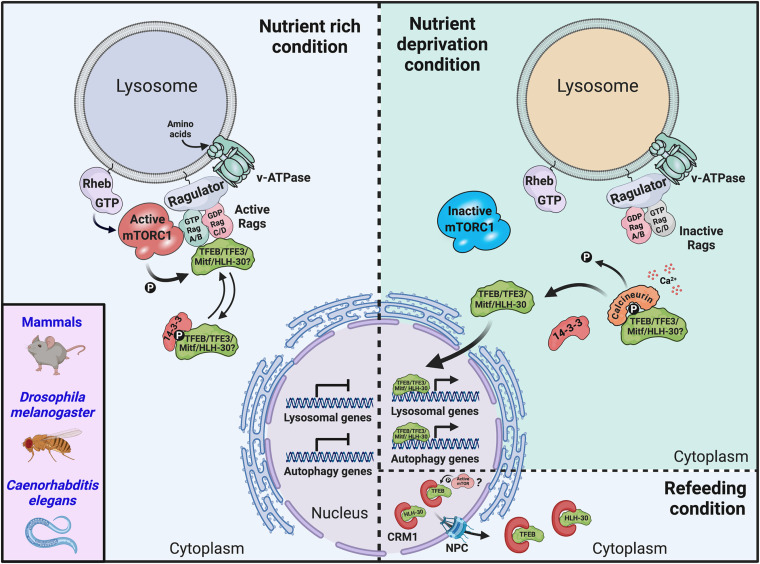
MiT/TFE activation is regulated in response to nutrient deprivation. Schematic representation of the mechanism of MiT/TFE transcription factor regulation by nutrient levels in the cell. In nutrient-rich condition, MiT/TFE proteins mammalian target of rapamycin complex 1 (mTORC1) are recruited to the lysosomal membrane through binding to active RagGTPases. Active mTORC1 phosphorylates MiT/TFE proteins at key residues that creates a binding site for the 14-3-3, which sequesters the transcription factors inactive in the cytosol. Under nutrient deprivation conditions, inactive RagGTPases lead to mTORC1 inactivation and MiT/TFE protein dissociation from 14-3-3 as a consequence of their dephosphorylation mediated by the calcium-dependent activation of calcineurin. Nuclear accumulation of MiT/TFE proteins mediates the activation of a transcriptional network that promotes autophagy, lysosomal biogenesis, and increased lysosomal degradation. Upon nutrient replenishment conditions, MiT/TFE proteins nuclear export is regulated by mTOR-dependent phosphorylation and binding to CRM1/Exportin-1. The question marks denote that there is no direct evidence available to support the indicated processes for some of the MiT/TFE family members. CRM1, chromosomal maintenance 1; NPC, nuclear pore complex; P, phosphorylation; Rheb, Ras homolog enriched in brain; v-ATPase, vacuolar-type H^+^-ATPase.

The subcellular localization and activity of TFEB and TFE3 are tightly controlled. Under nutrient-rich conditions, these transcription factors are recruited to the lysosomal surface by direct binding to active heterodimeric Rag GTPases (Martina and Puertollano, 2013); at the lysosome, active mTORC1 phosphorylates TFEB and TFE3 at several residues including serines 211 and 321, respectively (Martina et al., 2012; Roczniak-Ferguson et al., 2012; Settembre et al., 2012). This phosphorylation occurs through a substrate-specific mechanism (Napolitano et al., 2020). Phosphorylated TFEB and TFE3 are sequestered in the cytosol by binding to the chaperone-like protein 14-3-3 and remain inactive (Martina et al., 2012; Roczniak-Ferguson et al., 2012). Conversely, starvation results in Rag GTPases and mTORC1 inactivation, promoting TFEB and TFE3 dephosphorylation, and their dissociation from 14-3-3. This sequence of events promotes nuclear translocation and activation of TFEB and TFE3, leading to the upregulation of genes involved in lysosomal biogenesis and autophagy (Martina et al., 2012; Roczniak-Ferguson et al., 2012; Settembre et al., 2012). When the nutrients are replenished, TFEB nuclear export is modulated by mTORC1-dependent phosphorylation at serines 142 and 138; this phosphorylation is required for the recognition of TFEB nuclear export signal by chromosomal maintenance 1 (CRM1)/Exportin-1 (Napolitano et al., 2018). The silencing of *xpo-1*, the *C. elegans* ortholog of human Exportin-1, promotes the nuclear accumulation of HLH-30 in the worm (Silvestrini et al., 2018), suggesting that the nuclear exit control mechanism is conserved among species (Figure 3 and Table 1). A similar nutrient-dependent regulatory mechanism has been described for *D. melanogaster*, in which Mitf cytoplasmic retention/inactivation is mediated by TORC1 phosphorylation and subsequent 14-3-3 interaction. Mitf nuclear translocation/activation promotes the upregulation of all v-ATPase subunit genes, leading to the increase in the activity of this vacuolar ATP-dependent proton pump that negatively controls Mitf function (Zhang et al., 2015). Altogether, these observations highlight an evolutionarily conserved nutrient-responsive machinery dedicated to the control of MiT/TFE protein activation.

**TABLE 1:** Summary of functions and cellular processes associated with MiT/TFE proteins.

Protein	Functions	Processes	References
***C. elegans***			
HLH-30	Transcriptional response to oxidative, thermal, and proteotoxic stress and regulation of detoxifying enzyme expression.	Survival and life span extension.	Kenyon, 2010; Leiser et al., 2015; Bennett et al., 2017; Kumsta et al., 2017; Lee and Mylonakis, 2017; Lin et al., 2018; Liu et al., 2020
	Upregulation of SKN-1.	Increase in mitochondrial biogenesis.	Mansueto et al., 2017; Palikaras et al., 2017
	Regulation of lysosomal lipases and vitellogenins, response to glucose exposure.	Lipid mobilization and homeostasis, metabolic adaptation in response to food availability, cell death.	O’Rourke and Ruvkun, 2013; Harvald et al., 2017; Palikaras et al., 2017; Murphy et al., 2019
	Regulation of immune and auto-lysosomal gene upregulation, xenography.	*S. aureus* and Gram-positive bacterial infection.	Visvikis et al., 2014; Najibi et al., 2016; Chen H. D. et al., 2017; El-Houjeiri et al., 2019; Li et al., 2019
	Adult reproductive quiescence.	Life span extension.	Gerisch et al., 2020
	Protein aggregate formation.	Aging and neurodegeneration.	Kim et al., 2016; Butler et al., 2019
	Upregulation of autophagy and lysosomal genes.	Longevity.	Lapierre et al., 2013; Nakamura et al., 2016; Lin et al., 2018; Silvestrini et al., 2018; Liu et al., 2020
***D. melanogaster***			
Mitf	Autophagosome biogenesis and endo-lysosomal compartment.	Wing disk tissue formation.	Tognon et al., 2016
	Activation of autophagy and increase in lysosomal activity.	Decrease in lipid droplet size.	Bouche et al., 2016
	Upregulation by lysosomal dysfunction and autophagy block.	Compensatory mechanism in a Gaucher disease model.	Kinghorn et al., 2016
***D. rerio***			
Tfeb	Notochord vacuole formation.	Vertebra development.	Ellis et al., 2013; Sun et al., 2020
	Expansion of lysosomal compartment.	Microglial cells.	Gan et al., 2019
	Repression of myelinization in oligodendrocytes.	Neurodegeneration.	Meireles et al., 2018
**Mammals**			
TFEB/TFE3	Upregulation of genes involved in lysosomal biogenesis and autophagy.	Nutrient deprivation.	Sardiello and Ballabio, 2009; Sardiello et al., 2009; Settembre et al., 2011, 2012; Martina et al., 2012, 2014b; Roczniak-Ferguson et al., 2012; Martina and Puertollano, 2013; Medina et al., 2015
	TFEB inactivation by CRM1/Exportin-1.	Nutrient replenishment.	Napolitano et al., 2018
	Regulation at transcriptional level, autoregulatory-feedback loop and proteasomal degradation by STUB1.	Autoregulation.	Settembre et al., 2013; Ghosh et al., 2015; Sha et al., 2017
	Upregulation of lysosomal, autophagy or apoptotic genes induced by oxidative stress, ER stress, mitochondrial dysfunction or genotoxic stress. mTORC1-dependent and -independent mechanism.	Response to different stress conditions.	Medina et al., 2011; Palmieri et al., 2011; Settembre et al., 2011; Spampanato et al., 2013; Martina et al., 2014b, 2016; Zhang et al., 2016; Fernandez-Mosquera et al., 2017; Leow et al., 2017; Brady et al., 2018a; Martina and Puertollano, 2018; Pisonero-Vaquero et al., 2020; Zhang et al., 2020
	Induction of mitochondrial biogenesis and mitophagy.	Mitochondria biogenesis and quality control.	Settembre et al., 2013; Nezich et al., 2015; Salma et al., 2015; Mansueto et al., 2017; Erlich et al., 2018; Kim et al., 2018; Contreras et al., 2020; Popov, 2020
	Lipophagy and lipolysis induction. Obesity prevention and reversal.	Lipid catabolism.	Settembre et al., 2013; Emanuel et al., 2014; Pastore et al., 2017
	Glycogen synthesis and hyperglycemia reduction.	Transcriptional control of the insulin pathway and glucose metabolism.	Nakagawa et al., 2006; Iwasaki et al., 2012; Salma et al., 2015; Mansueto et al., 2017; Pastore et al., 2017
	Adipose tissue regulation. Induction or reduction of WAT browning, autophagy and lipolysis.	Regulation of whole-body metabolism.	Fujimoto et al., 2013; Wada et al., 2016; Chen L. et al., 2017; Evans et al., 2019; Huber et al., 2019
	Upregulation of lysosomal genes, cytokines and inflammatory genes.	Pathogenic infection and immune response.	Visvikis et al., 2014; Campbell et al., 2015; Najibi et al., 2016; Ouimet et al., 2016; Pastore et al., 2016; Nabar and Kehrl, 2017; Wu et al., 2017; Brady et al., 2018b; Singh et al., 2018; El-Houjeiri et al., 2019; Li et al., 2019; Irazoqui, 2020
	TFE3 gain-of-function induces developmental syndrome.	Development.	Villegas et al., 2019; Diaz et al., 2020
**Mammals**			
TFEB/TFE3	Reduction of stem cell differentiation by induction of a quiescent state	Stem cell differentiation	Betschinger et al., 2013; Young et al., 2016; Hu et al., 2020; Kobayashi et al., 2019
	Myelinization repression	Neurodegeneration	Meireles et al., 2018
	Regulation of cell cycle transition and proliferation	Cell cycle	Malumbres and Barbacid, 2009; Young et al., 2016; Doronzo et al., 2019; Kobayashi et al., 2019; Pastore et al., 2020; Pisonero-Vaquero et al., 2020; Yin et al., 2020
	Involvement in pancreatic adenocarcinomas, renal carcinoma development, cellular migration, and tumorigenesis	Cancer	Levy et al., 2006; Kauffman et al., 2014; Giatromanolaki et al., 2015; Perera et al., 2015, 2019; Calcagni et al., 2016; Raben and Puertollano, 2016; Bretou et al., 2017; Di Malta et al., 2017; Sakamoto et al., 2018; Doronzo et al., 2019; Eichner et al., 2019; Wylie et al., 2019
MITF	Regulation of expression of genes involved in eye differentiation, epidermis pigmented cells, and deafness	Development	Hallsson et al., 2004; Curran et al., 2010; Liu et al., 2010; Goding and Arnheiter, 2019

*CRM1, chromosomal maintenance 1; ER, endoplasmic reticulum; HLH-30, helix-loop-helix transcription factor 30; mTORC1, mammalian target of rapamycin complex 1; SKN-1, skinhead 1; STUB1, STIP1 homology and U-box-containing protein 1; WAT, white adipose tissue.*

Also, under starvation conditions, activation of the protein phosphatase calcineurin (induced by localized calcium release from lysosomes) results in TFEB dephosphorylation at critical serine residues, which regulates its nuclear localization and activity (Medina et al., 2015). In addition, TFEB and TFE3 themselves are controlled at the transcriptional level (Ghosh et al., 2015); for example, TFEB can regulate its own expression through a starvation-induced autoregulatory-feedback loop (Settembre et al., 2013). Yet another mechanism of TFEB regulation involves its proteasomal degradation through preferential targeting of inactive TFEB by STIP1 homology and U-box-containing protein 1 (STUB1), a chaperone-dependent E3 ubiquitin ligase (Sha et al., 2017). Moreover, phosphorylation at key residues by kinases, such as mitogen-activated protein kinase 1 (MAPK1), mitogen-activated protein kinase kinase kinase kinase 3 (MAP4K3), glycogen synthase kinase (GSK)3β, v-Akt murine thymoma viral oncogene homolog (AKT), and c-Abl (Settembre et al., 2011; Li et al., 2016; Palmieri et al., 2017; Hsu et al., 2018; Contreras et al., 2020), as well as other posttranslational modifications such as acetylation, SUMOylation, and oxidation (Miller et al., 2005; Zhang et al., 2018; Wang et al., 2019; Wang Y. et al., 2020) may also contribute to the regulation of TFEB and TFE3 activity in response to different stimuli.

## MiT/TFE Transcription Factors Are Activated in Response to Various Stress Conditions

**FIGURE 4:**
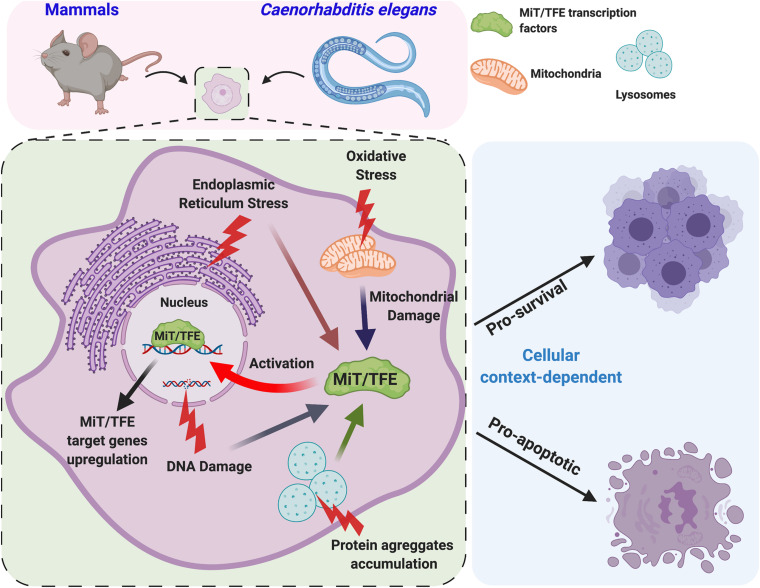
Various stress conditions activate MiT/TFE transcription factors. In *C. elegans* and mammalian cells, different internal and external stress conditions such as DNA damage, protein aggregate accumulation in endo-lysosomal compartments, endoplasmic reticulum (ER) stress, oxidative stress, and mitochondrial dysfunction lead to the activation of MiT/TFE transcription factors. In response to stress, these transcription factors upregulate the expression of a gene network involved in lysosomal function and autophagy flux to promote organismal survival. However, under severe stress conditions, MiT/TFE transcription factors may lead to the expression of apoptotic genes, promoting cell death.

As discussed in the previous section, MiT/TFE factors control cellular adaptation response to nutrient availability in an mTORC1-dependent manner; however, emerging evidence points to their broader role in transcriptional control of the processes caused by different stresses or physiological aging, in some cases, through a mechanism independent of mTORC1 activity (Figure 4 and Table 1).

*In vitro* experiments performed in mammalian cells have shown that both oxidative stress (induced by H_2_O_2_, chloramine T, and sodium arsenite) and ER stress due to the accumulation of unfolded proteins or perturbation of ER homeostasis (Martina et al., 2016; Zhang et al., 2016, 2020; Leow et al., 2017; Martina and Puertollano, 2018) promote nuclear translocation and activation of TFEB and TFE3 where they upregulate the expression of lysosomal and autophagy genes to counter detrimental effects (Zhang et al., 2016, 2020; Leow et al., 2017). However, in case of a severe and prolonged stress, TFEB and TFE3 may also regulate the expression of apoptotic genes (Martina et al., 2016).

An important outcome of these studies is the existence of multiple molecular mechanisms leading to MiT/TFE activation in addition to mTORC1-dependent regulation. These may include a more general action of calcineurin (Martina et al., 2016; Zhang et al., 2016), as well as a context-specific activity of protein phosphatase 2A (PP2A) and PKR-like endoplasmic reticulum kinase (PERK)/splice X-box binding protein 1 (sXBP1), the proteins that are involved in oxidative stress response and unfolded stress response, respectively (Martina et al., 2016; Martina and Puertollano, 2018; Zhang et al., 2020). In addition, mitophagy induction results in MiT/TFE protein activation through a mechanism that requires the mitochondrial kinase PTEN-induced kinase (PINK), the E3 ubiquitin ligase parkin, and the autophagy proteins ATG9A and ATG5 (Nezich et al., 2015). TFEB and TFE3 are also activated in response to genotoxic stress through a p53 and mTORC1-dependent mechanism (Brady et al., 2018a), leading to cell cycle arrest or apoptosis depending on the severity of the DNA damage (see *Cell Proliferation, Differentiation, and Tumorigenesis* section).

The role of MiT/TFE proteins in the adaptation to stress also has been observed in invertebrates. In *C. elegans*, HLH-30 is activated by both oxidative and heat stress to promote a transcriptional response required for survival and life span extension (Lin et al., 2018). Under these conditions, the complex transcriptional response of HLH-30 appears to be synergized with DAF-16 [the conserved forkhead transcription factor and the sole ortholog of human forkhead box O (FOXO)]. HLH-30 can physically interact with DAF-16 to form a complex in a context-dependent manner to co-regulate a combinatorial transcriptional response to oxidative stress and promote longevity, whereas heat stress response and developmental decisions can be regulated independently of each other (Kenyon, 2010; Lin et al., 2018).

In addition to external factors, stressful conditions may originate inside the cell. In this regard, it is worth reiterating that mitochondria represent the major source of reactive oxygen species (ROS) production. This is not only due to the activity of the mitochondrial respiratory chain complexes but also owing to the perturbation of mitochondrial morphology, which are caused by mutations in the proteins of mitochondrial network dynamics as well as metabolic enzymes. Indeed, activation of HLH-30 has been observed in *C. elegans* models characterized by the lack of transaldolase-1 (an enzyme of the pentose phosphate pathway) (Bennett et al., 2017) or fission and fusion proteins (Liu et al., 2020). Consequently, the expression of genes encoding autophagy/lysosomal proteins and detoxifying enzymes is enhanced, resulting in a pro-longevity effect (Bennett et al., 2017; Liu et al., 2020).

Moreover, HLH-30 is activated in response to age- and stress-related accumulation of granulins in the endo-lysosomal compartment, leading to the impairment of lysosomal function (Butler et al., 2019). Also, in both *C. elegans* and mammalian cell models of aggregation-prone proteins, HLH-30 and TFEB activation are regulated by reduced glutathione and the activity of glutathione reductase (GSR-1) (Guerrero-Gomez et al., 2019).

Thus, MiT/TFE factors may be generally defined as vigilant sensors responsible for transcriptional rearrangements that allow cellular adaptation to internal and external stresses. The conservation of these processes across species underscores the possibility of using different animal models to dissect the contribution of MiT/TFE transcription factors in a variety of diseases.

## Regulation of the Biogenesis of Lysosomes, Autophagosomes, and Mitochondria in Response to Stress

**FIGURE 5:**
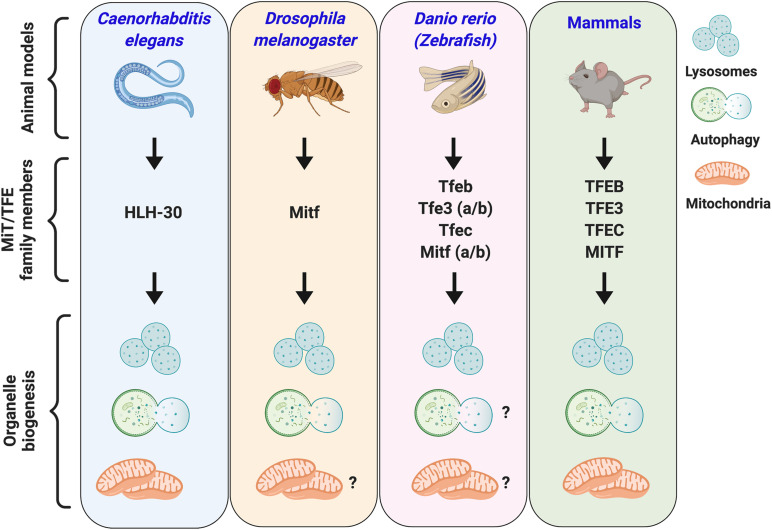
MiT/TFE transcription factors modulate lysosome, autophagosome, and mitochondrial biogenesis in response to stress. Summary of organelle biogenesis induction in different animal models controlled by the MiT/TFE proteins. In most of the animal models, the activation of the MiT/TFE proteins in response to stress conditions induces an upregulation of genes involved in lysosomal biogenesis, autophagy, and mitochondrial biogenesis. The question marks signify that there are no data available to support organelle biogenesis induction in the indicated animal models; however, it is likely that these processes may take place based on the highly conserved functions between the different MiT/TFE family members across species.

During organelle biogenesis, new membrane-bound compartments are formed (Mullock and Luzio, 2005) through a process that is transcriptionally regulated in response to cellular needs and/or stress conditions (Dhaunsi, 2005; Martina et al., 2014a; Yang et al., 2018; Ballabio and Bonifacino, 2020). The MiT/TFE transcription factors, and especially TFEB and TFE3, play key roles in this process (Sardiello and Ballabio, 2009; Sardiello et al., 2009; Settembre et al., 2011; Martina et al., 2014b) by upregulating the expression of hundreds of genes involved in autophagosome and lysosome formation and function in response to stress conditions (Sardiello et al., 2009; Palmieri et al., 2011; Settembre et al., 2011) (Figure 5 and Table 1).

The enhancement of lysosomal biogenesis and autophagy is needed for proper cellular response to stress. Indeed, activation of TFEB and TFE3, first described under nutrient deprivation (to provide energy), was also observed under other stress conditions, such as oxidative stress, mitochondrial dysfunction, and accumulation of unfolded proteins in the ER, in these cases to rid the cells of aberrantly accumulated substrates and damaged organelles (Medina et al., 2011; Palmieri et al., 2011; Settembre et al., 2011; Spampanato et al., 2013; Martina et al., 2014b, 2016; Nezich et al., 2015; Fernandez-Mosquera et al., 2017; Martina and Puertollano, 2018).

Several studies in invertebrate models have demonstrated that these molecular mechanisms have been evolutionarily conserved. HLH-30 and Mitf, the single MiT/TFE family orthologs found in *C. elegans* and *D. melanogaster*, respectively, undergo regulatory processes similar to those described for TFEB and TFE3 in mammals, namely, their activation and translocation into the nucleus upon different stresses (e.g., starvation, heat stress, pathogen infection) to regulate the expression of genes involved in autophagy and lysosomal biogenesis (Lapierre et al., 2013; O’Rourke and Ruvkun, 2013; Settembre et al., 2013; Visvikis et al., 2014; Zhang et al., 2015; Bouche et al., 2016; Najibi et al., 2016; Tognon et al., 2016; Lin et al., 2018).

The control of autophagosome biogenesis and endo-lysosomal compartments is necessary not only for survival in the face of biological challenges but also for maintenance of homeostasis and development. For instance, Mitf is involved in the formation of the wing disk tissue in flies (Tognon et al., 2016), while Tfeb in *Danio rerio* (zebrafish) promotes the generation of lysosome-related vacuoles in the notochord of zebrafish embryos, allowing for the proper morphogenesis of the vertebrae (Ellis et al., 2013; Sun et al., 2020) (see *Organism Development, Longevity, and Survival to Stress* section). Also, activation of TFEB and expansion of the lysosomal compartment were observed in microglial cells of zebrafish mutants, although physiological significance of this finding is not clear (Gan et al., 2019).

The elimination of malfunctioning organelles by autophagy and their replacement with newly generated ones is a process of great pathophysiological relevance. The mitochondrial quality control mechanism involves degradation of damaged mitochondria by the concerted action of PINK and parkin proteins, activation of members of the MiT/TFE family, and induction of mitophagy (Nezich et al., 2015). Besides their role in mitochondrial clearance, MiT/TFE transcription factors (as well as several others) regulate mitochondrial biogenesis in a complex process that requires the expression of ∼1,000 proteins and leads to an increase in both mitochondrial mass and number (Popov, 2020). Mitochondrial biogenesis occurs in response to a variety of stimuli. TFEB and TFE3 regulate the expression of multiple mitochondrial genes under conditions of energy demand (such as, for example, acute exercise or nutrient deprivation) (Settembre et al., 2013; Salma et al., 2015; Erlich et al., 2018) or environmental insults (Kim et al., 2018), and they do so either by direct activation of the mitochondrial transcriptional program (Mansueto et al., 2017) or by modulation of peroxisome proliferator-activated receptor-gamma coactivator 1 alpha (PGC-1α), the master regulator of mitochondrial biogenesis (Settembre et al., 2013; Salma et al., 2015; Erlich et al., 2018).

Most studies on the involvement of MiT/TFE family in the regulation of mitochondrial biogenesis have been performed in mammalian models. However, the ability of HLH-30 to induce autophagy and lysosomal biogenesis in response to mitochondrial dysfunction (Bennett et al., 2017; Liu et al., 2020) and to upregulate the expression of skinhead-1 [SKN-1; the nematode ortholog of the transcription factor nuclear factor erythroid 2-related factor 2 (NRF-2)], which in turn enhances mitochondrial biogenesis under stress conditions (Mansueto et al., 2017; Palikaras et al., 2017), suggests that the role of MiT/TFE proteins in the maintenance of mitochondrial homeostasis and organism adaptation to stress dates all the way back.

## Modulation of Key Metabolic Pathways in Response to Energy Needs

**FIGURE 6:**
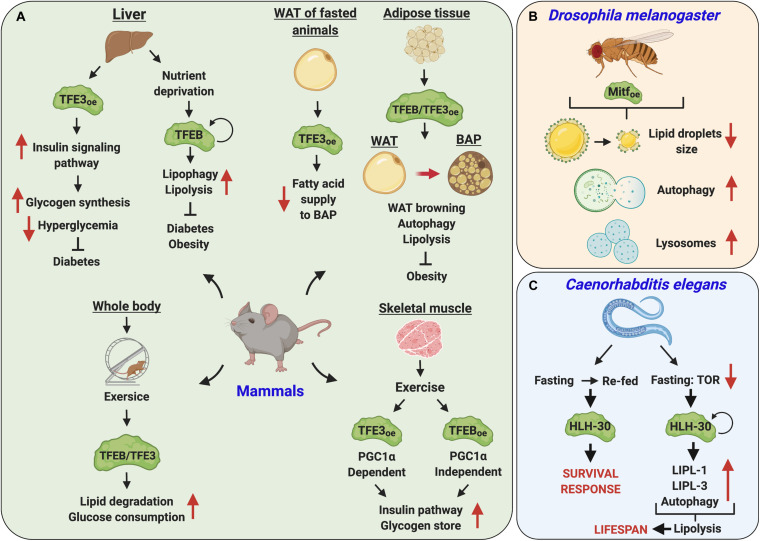
Key metabolic pathways are regulated by MiT/TFE transcription factors under energy demand conditions. **(A)** MiT/TFE proteins regulate cellular energy state and metabolism in mammals. Activated and overexpressed (oe) TFEB and TFE3 regulate lipid metabolism and insulin signaling pathways in metabolic organs such as liver, skeletal muscle, and adipose tissue. By upregulating genes involved in autophagy/lipophagy, insulin signaling, and degradation of lipids and in the utilization of glucose to promote glycogen synthesis, these transcription factors play an essential role in reducing diabetes and obesity in mice. **(B)** In *D. melanogaster*, under nutrient deprivation conditions, Mitf overexpression (oe) induces a reduction in lipid droplet size through the activation of autophagy and increase in lysosomal activity. **(C)** In fasted *C. elegans*, helix-loop-helix transcription factor 30 (HLH-30) upregulates the expression of lysosomal lipases (LIPL-1 and LIPL-3) and autophagy genes controlling lipid mobilization *via* lipolysis. Also, the activation of HLH-30 is central for the survival response to conditions of fasting-refeeding *via* TOR regulation. ↺ indicates autoregulatory loop. ↑↓ indicate upregulation and downregulation, respectively. BAT, brown adipose tissue; WAT, white adipose tissue.

In response to environmental challenges, such as food deprivation, multicellular organisms adapt their metabolic signaling pathways to convey the energy needs to survive. The transcriptional regulation of these pathways is fundamental for the sustained response to stress, and the dysfunction of these fine-tuned processes entails dire consequences. In humans, alterations in energy homeostasis and metabolic imbalances are the cause of several disorders such as diabetes, obesity, and metabolic syndrome (Ghanemi et al., 2018). A growing body of evidence has positioned the MiT/TFE proteins as critical regulators of cellular energy state and metabolism (Figure 6 and Table 1).

These proteins appear to play important and context-specific roles in the liver, skeletal muscle, and adipose tissue. In the liver of fasted mice, TFEB is required for lipid catabolism, which is upregulated through PGC1-α and peroxisome proliferator-activated receptor-1-alpha (Ppar1-α), both master regulators of lipid metabolism in this tissue (Settembre et al., 2013). TFEB upregulates the expression of lysosomal acid lipase, the enzyme responsible for the hydrolysis of cholesteryl esters and triglycerides in lysosomes (Settembre et al., 2013; Emanuel et al., 2014). Remarkably, the increase in TFEB activity in the liver can prevent and even reverse obesity, a component of the metabolic syndrome in humans (Settembre et al., 2013).

Analysis of TFE3 provided further insights into the role of MiT/TFE transcription factors in the regulation of energy state and metabolism. TFE3 was shown to transcriptionally control insulin signaling pathway in mouse liver, mainly through the insulin receptor substrate 2 (IRS2)/phosphoinositide 3-kinase (PI3K)/AKT axis. Consequently, TFE3 overexpression improved glucose metabolism by promoting glycogen synthesis and reducing hyperglycemia in diabetic mice (Nakagawa et al., 2006).

The action of insulin is relevant not only in the liver but also in other tissues, such as skeletal muscle, where TFEB and TFE3 are required for the upregulation of genes involved in insulin signaling and glucose metabolism in animals subjected to physical exercise. Interestingly, the molecular mechanisms underlying the activities of these two transcription factors do not always match, as indicated, for example, by a PGC1-α-dependent and -independent action of TFE3 and TFEB, respectively (Iwasaki et al., 2012; Salma et al., 2015; Mansueto et al., 2017). Furthermore, TFEB cooperatively participates with TFE3 in the regulation of glucose and lipid homeostasis in the adaptive response to physical stress (Pastore et al., 2017).

For a long time, adipose tissue has been defined as a passive fat reservoir. Instead, it is now believed to be critical in the regulation of the whole-body energy metabolism, with the white adipose tissue (WAT) providing lipid substrates to generate energy and the brown adipose tissue (BAT) playing a role in thermogenesis by utilizing the degraded lipids from WAT to stimulate mitochondrial β-oxidation (Kahn et al., 2019). In adipose tissue, TFEB activity appears to have a health-promoting effect. Its activation is required to promote autophagy and lipolysis as a part of an acetyl-coenzyme A sensing mechanism (Huber et al., 2019). TFEB overexpression was shown to protect animals from diet-induced obesity by upregulating genes that improve metabolic rate, reduce adiposity, and induce white fat browning and cold tolerance (Evans et al., 2019). The role of TFE3 in adipose tissue is still not fully understood. Its overexpression in WAT of fasted animals downregulates genes involved in lipolysis resulting in the inhibition of fatty acid supply to BAT, and thus reducing thermogenesis (Fujimoto et al., 2013). On the other hand, the adipocyte-specific deletion of folliculin (FLCN) attenuates TFE3 inhibition by mTORC1, leading to the induction of the transcriptional coactivator PGC-1β and upregulation of genes that participate in WAT browning (Wada et al., 2016).

Energy metabolism is also regulated by hormonal signaling. In particular, the fasting-induced hormone fibroblast growth factor 21 (FGF21), a master regulator of energy homeostasis during nutritional stress, promotes lipolysis, fatty acid oxidation, and gluconeogenesis (Kharitonenkov et al., 2005). Under nutrient deprivation, TFEB is activated by FGF21, leading to the upregulation of genes involved in lysosomal biogenesis, autophagy, and lipid metabolism in the liver, skeletal muscle, and adipose tissue. These findings signify a coordinated and interorgan action of MiT/TFE transcription factors in addition to their organ-specific activity (Chen L. et al., 2017).

The cardinal role of TFEB and TFE3 in the regulation of metabolic pathways is conserved through evolution, as demonstrated by several studies performed in *C. elegans.* HLH-30 upregulates the expression of autophagic genes and lysosomal lipases to promote lipid mobilization; HLH-30 also controls the expression of vitellogenins (lipoproteins involved in lipid homeostasis) and prevents the accumulation of ectopic fat in response to fasting (O’Rourke and Ruvkun, 2013; Harvald et al., 2017; Palikaras et al., 2017). Accordingly, multi-omics analyses revealed that the functional loss of HLH-30 causes a profound alteration in metabolic pathways, particularly in lipoprotein metabolism in fasted animals (Harvald et al., 2017). HLH-30 mutant worms display a significant decrease in life span under nutrient deprivation conditions, further confirming the relevance of MiT/TFE transcription factor(s) in the metabolic adaptation response to food availability and survival (O’Rourke and Ruvkun, 2013; Settembre et al., 2013). In addition, the overexpression of Mitf in *D. melanogaster* resulted in a decrease in lipid droplets’ size in response to nutrient deprivation (Bouche et al., 2016).

Overall, the current data position TFEB-TFE3-HLH-30-Mitf as pivotal master regulators of lipid and energy metabolisms with an important contribution to cell survival in response to environmental insults across species. Although this field is rapidly expanding, many questions still remain unresolved.

## Regulation of Immune Response Against Pathogen Infection

The immune response is a central component of an effective defense mechanism that is tightly controlled during infection or injury, and its dysregulation may lead to pathological conditions, such as autoimmune diseases and oncogenesis, characterized by different inflammation states.

**FIGURE 7:**
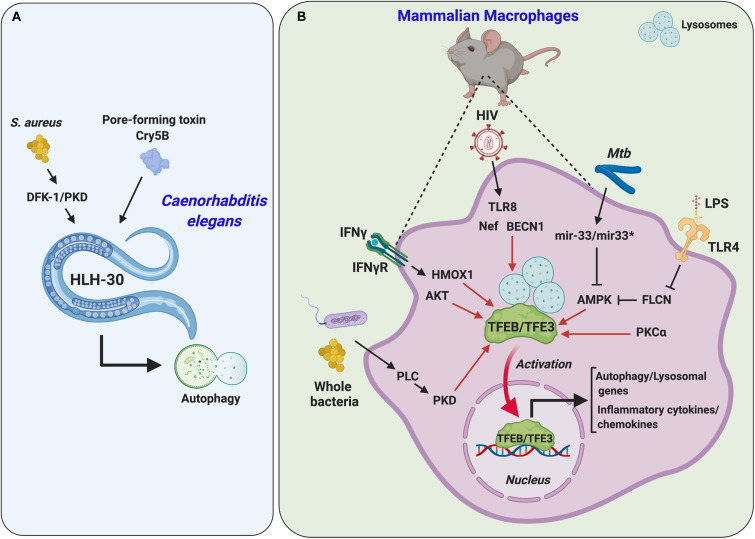
The stress response to pathogen infection and inflammation is modulated by MiT/TFE transcription factors. **(A)** In *C. elegans*, the activation of helix-loop-helix transcription factor 30 (HLH-30) is mediated by *S. aureus* and pore-forming toxin Cry5B. **(B)** In mammalian macrophages, TFEB and TFE3 activation is regulated by many different stimuli. Activated TFEB and TFEB3 upregulate the expression of genes involved in autophagy/lysosome-related processes and inflammatory cytokine/chemokine production. AKT, v-Akt murine thymoma viral oncogene homolog; AMPK, AMP-activated protein kinase; BECN1, beclin-1; FLCN, folliculin; HIV, human immunodeficiency virus; HMOX1, heme oxygenase 1; IFNγ, interferon γ; IFNγR, interferon γ receptor; LPS, lipopolysaccharide; Mtb, *M. tuberculosis*; Nef, negative regulatory factor; PKCα, protein kinase C alpha; PKD, protein kinase D; PLC, phospholipase C; TLR4, Toll-like receptor 4; TLR8, Toll-like receptor 8.

The relationship between the lysosome–autophagy pathway and the immune system is now well-established. Several studies have shown the involvement of MiT/TFE transcription factors in the control of innate immunity and inflammation and in the mechanism of host defense against pathogenic infections from simple metazoans to mammals (Figure 7 and Table 1). The important role of MiT/TFE factors in pathogen response was first demonstrated in *C. elegans*. Upon recognition of extracellular bacteria *Staphylococcus aureus*, HLH-30 activates and translocates into the nucleus to modulate the expression of genes involved in immune and lysosome–autophagy signaling (Visvikis et al., 2014). Furthermore, an *in vivo* reverse genetic screening in *C. elegans* revealed that D kinase family 1 (DKF-1), the ortholog of mammalian protein kinase D (PKD), is responsible for the activation of HLH-30 in response to pathogen infection (Najibi et al., 2016). A similar mechanism was described in mice, where a phospholipase C (PLC)–PKD axis and protein kinase C-alpha (PKC-α) activity along with TFEB activation in murine macrophages are required after pathogenic infection (Najibi et al., 2016), suggesting that this response is evolutionarily conserved.

In addition to *S. aureus*, the activation of HLH-30 and autophagy occur in animals fed on Gram-positive bacterial pore-forming toxins, known to be involved in xenophagy and membrane repair program in *C. elegans* intestinal cells (Chen H. D. et al., 2017). Taken together, these findings demonstrate the link between HLH-30-mediated autophagy and host defense system and suggest that the HLH-30-mediated response to infection is not pathogen-specific. These observations are further supported by studies showing that mammalian TFEB and TFE3 can be activated in macrophage cell lines by several Toll-like receptor (TLR) ligands (Visvikis et al., 2014; Pastore et al., 2016; Brady et al., 2018b) and upon exposure to lipopolysaccharide (LPS) (Li et al., 2019), leading to the transcriptional control of cytokines, chemokines, and other inflammatory genes, in addition to the upregulation of lysosomal genes (Visvikis et al., 2014; Pastore et al., 2016; Brady et al., 2018b; El-Houjeiri et al., 2019). Also, recent studies uncovered a regulatory mechanism involving the tumor suppressor protein FLCN, which confers resistance to various stress conditions *via* AMP-activated protein kinase (AMPK) regulation both in *C. elegans* and mammalian models (El-Houjeiri et al., 2019; Li et al., 2019). These data underscore the importance of the FLCN–TFEB/TFE3 pathway in the cellular immune response and pathogen resistance and highlight the evolutionary conservation of these processes.

The important role of TFEB in immune response is further emphasized by the observation that several pathogens modulate TFEB activity for their own benefit. This is the case of *Mycobacterium tuberculosis*, which downregulates the expression of TFEB and its target genes through upregulation of two microRNAs (Mir33 and Mir33^∗^) and inhibition of AMPK (Ouimet et al., 2016).

The immune system is essential for building up protective response not only against bacteria but also against viral infections. In this regard, several studies have proposed TFEB as a critical regulator of antiviral response in mammals. Transient activation of TFEB and autophagy was reported upon initial exposure of human macrophages to HIV infection through a mechanism involving TLR8 and Beclin-1. However, during later stages of viral replication, the interaction between HIV protein, Nef, and Beclin-1 promotes TFEB phosphorylation and inactivation, thus inhibiting autophagy (Campbell et al., 2015). In addition, influenza virus infection regulates phagocytosis by inactivating TFEB and AKT through the interferon (IFN)γ pathway (Wu et al., 2017). In contrast, an IFNγ–heme oxygenase 1 (HMOX1)-dependent mechanism of TFEB activation in murine RAW264.7 cells has been recently reported (Singh et al., 2018). These data indicate that the antiviral functions of MiT/TFE proteins are not straightforward.

The role of MiT/TFE proteins in modulating immune response to pathogen infection is in part linked to their ability to regulate the lysosome–autophagy pathway, and the mechanisms governing these processes seem evolutionarily conserved from lower metazoans to mammals. We refer the readers to several recent comprehensive reviews for additional discussion on the role of the MiT/TFE of transcription factors in innate immunity and inflammation (Nabar and Kehrl, 2017; Brady et al., 2018b; Irazoqui, 2020).

## Organism Development, Longevity, and Survival to Stress

**FIGURE 8:**
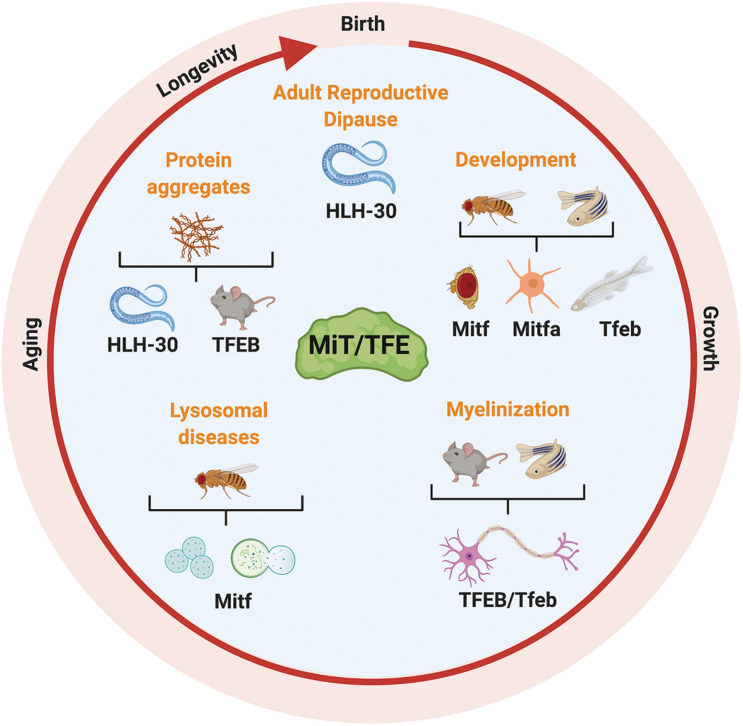
Longevity and survival are promoted by MiT/TFE transcription factors. The MiT/TFE transcription factors control longevity and survival, inducing correct development, promoting healthy aging, and preventing cognitive decline. In *C. elegans*, helix-loop-helix transcription factor 30 (HLH-30) regulates life span extension in starvation-induced adult reproductive diapause conditions. During development, MiT/TFE proteins regulate differentiation of the eye and epidermis pigmented cells in *D. melanogaster* and proper vertebra formation during embryogenesis in zebrafish notochord. In addition, MiT/TFE proteins repress myelinization and induce autophagy and lysosome integrity to face aging neuronal decline in neurodegenerative lysosomal storage disorders and aberrant protein accumulation.

The development of genetic tools and their applications to animal models revealed that MiT/TFE transcription factors are involved in the control of growth and development, aging, and death (Figure 8 and Table 1). In particular, simple organisms like the *C. elegans* nematode worm represent a powerful model to study the role of MiT/TFE proteins in longevity.

Several studies in this organism showed that HLH-30 and the upregulation of its target genes involved in autophagy and lysosomal activity are required in many longevity models (e.g., tor, eat-2, daf-2, clk, rsks-1, and glp-1, mitochondrial mutants) (Lapierre et al., 2013; Liu et al., 2020). Interestingly, “fine-tuning” in these models is achieved by upregulating distinct autophagic genes in different mutants, indicating a pivotal role of HLH-30 in longevity (Lapierre et al., 2013). Accordingly, the inhibition of the nuclear export machinery, which enhances HLH-30 activity and autophagy, promotes life span extension in *C. elegans* (Silvestrini et al., 2018). In addition, upregulation of HLH-30-mediated autophagy makes worms more resistant to thermal and proteotoxic stress (Kumsta et al., 2017), whereas the HLH-30-dependent upregulation of the lysosomal acid lipase-2 (Lipl-2) allows *C. elegans* larvae to survive under starvation conditions. Notably, Lipl-2 is not only critical for providing energy but also for the generation of signaling molecules, thus coupling the lysosomal nutrient sensing system to TOR reactivation and TOR-mediated growth after refeeding (Murphy et al., 2019). Of note, the effect of MiT/TFE proteins on the life span is synergized by the association with DAF-16/FOXO transcription factor in a stimulus-dependent manner (Lin et al., 2018) and by a mutual regulation with the Myc and Mondo-Like (MML)1/Max-Like (MXL)2/MondoA/B transcriptional complex under germline precursors removal and starvation (Nakamura et al., 2016). All these observations underscore the important role of the MiT/TFE proteins in the modulation of the longevity pathways.

In addition to the autophagy–lysosome functions, other gene networks may be modulated by HLH-30 to control the life span. It has been recently demonstrated that HLH-30 regulates life span extension in worms that have undergone adult reproductive quiescence induced by fasting. This process drives the upregulation of genes involved in TOR signaling, mitochondrial dynamics, energy sensing, and an extended HLH network of transcriptional regulators such as MLX-2 and MLX-3. Furthermore, HLH-30 totally accounts for this process, as suggested by the 90% mortality rate observed in HLH-30 mutants under this condition (Gerisch et al., 2020).

HLH-30 also contributes to longevity and survival through the brain–gut crosstalk, resulting in the higher expression of the detoxifying enzyme flavin-containing monooxygenase (FMO)-2 (Leiser et al., 2015; Bennett et al., 2017) or ins-11, a neuropeptide that represses the aversion to pathogenic food (Lee and Mylonakis, 2017). Notably, depending on the cellular context, HLH-30 may also induce cell death and shorten the life span, as was demonstrated in *C. elegans* exposed to a high-glucose diet (Franco-Juarez et al., 2018). Although confirming this dual role in higher animals is a challenging task, the identification of genic counterparts and experiments in mammalian cells suggested that these mechanisms might be conserved (Lapierre et al., 2013; Leiser et al., 2015; Lin et al., 2018; Silvestrini et al., 2018).

The members of the MiT/TFE family are involved in regulating organism development. Tfeb was shown to facilitate proper vertebra formation by promoting the biogenesis of lysosome-related vacuoles during embryogenesis in the zebrafish notochord. The upstream molecular mechanism involves dstyk-mediated inhibition of mTOR, resulting in the activation of zebrafish Tfeb. In fact, dstyk mutants show a severe scoliosis phenotype that may be partially rescued by the administration of the selective mTOR inhibitor, Torin-1 (Sun et al., 2020). The direct role of TFEB in vertebra formation has not been confirmed in mammals because of the embryonic lethality (caused by alterations in placental vascularization) of TFEB knockout (KO) mice (Steingrimsson et al., 1998). However, excessive activation of mTORC1 signaling in chondrocytes was shown to result in congenital deformity of the spinal cord in mice (Yang et al., 2017). Notably, scoliosis phenotypes can be partially rescued by the mTORC1 inhibitor rapamycin (Yang et al., 2017).

In mice, MITF-regulated genes were shown to be involved in the differentiation of the eye and epidermis pigmented cells (Hallsson et al., 2004; Curran et al., 2010; Liu et al., 2010); the molecular mechanisms of these processes are extensively discussed (Hallsson et al., 2004; Goding and Arnheiter, 2019).

MiT/TFE proteins also play an important role in human development. A large body of evidence indicates that germline mutations in human MITF gene are mainly associated with pigmentation abnormalities and deafness (Goding and Arnheiter, 2019). Furthermore, recent works show that some gain-of-function TFE3 mutant alleles, which arise by amino acid substitutions affecting the Rag binding region of TFE3, are associated in humans with the X-linked dominant developmental syndrome with clinical manifestation of severe intellectual disability, coarse facial features, and Blaschkoid pigmentary mosaicism (Villegas et al., 2019; Diaz et al., 2020). In addition, embryonic stem cell transition to differentiated states is negatively regulated by TFE3 activation, which is controlled by the FLCN/folliculin-interacting protein (Fnip)1/Fnip2 complex (Betschinger et al., 2013). Furthermore, TFE3 activation is responsible for the conversion of primed human pluripotent stem cells to the naive state when mTOR is transiently inhibited (Hu et al., 2020). These findings suggest a role for TFE3 as a repressor of the genes required for the fate specification of pluripotent embryonic stem cells, a process that is prevented by pro-differentiation signaling pathway regulated by FLCN and a non-canonical RagGTPases mechanism during normal development.

The role of MiT/TFE transcription factors during development may, however, be more subtle as shown in *D. melanogaster* (Tognon et al., 2016). The fly Mitf indirectly modulates endosomal network and function (to support Notch signaling in the wing imaginal disk) through the regulation of the expression of specific V-ATPase pump subunits (Tognon et al., 2016).

Living organisms are continually exposed to a vast variety of stresses across their life span. The defense mechanisms and cellular processes underlying normal development promote healthy aging, limiting the occurrence of cognitive decline (Labbadia et al., 2017; El Assar et al., 2020). As such, neurodegeneration, a progressive age-related disease, is mainly associated with the accumulation of altered proteins and organelles (Lim and Yue, 2015). Lysosomal dysfunction, caused by the buildup of neurotoxic proteins or undigested materials, has been linked to neurodegenerative diseases (Kinghorn et al., 2016; Butler et al., 2019). Studies ranging from worms to mammals have demonstrated that the maintenance of autophagy and lysosome integrity by MiT/TFE target genes represents a universal mechanism to address age-related neuronal decline and to fight noxious factors (Kim et al., 2016; Butler et al., 2019). For example, improved lysosomal homeostasis by HLH-30 overexpression in worms slows down age-dependent SNCA/α-synuclein aggregate formation and transmission in neurons and pharynx muscle (Kim et al., 2016).

On the other hand, the compensatory upregulation of fly Mitf expression, induced by lysosomal dysfunction and autophagy block in the brain of a *D. melanogaster* model of Gaucher disease (GD), was unable to overcome the autophagy impairment (Kinghorn et al., 2016). Likewise, the ectopic expression of TFEB in induced pluripotent stem cell (iPSC)-derived neuronal cells from patients with GD was unable to restore the lysosomal function and clear the autophagosome accumulation (Awad et al., 2015). These data should be a cautionary note indicating that the functions of MiT/TFE proteins depend on the cellular context.

In higher organisms, the importance of MiT/TFE proteins in neurodegeneration goes beyond their lysosome-sustaining function and reflects a broader regulation capability of these transcription factors. Indeed, Tfeb has been shown to repress myelinization of oligodendrocytes in rraga and lamtor4 zebrafish embryo mutants (Meireles et al., 2018). Consistent with this, a higher amount of myelin basic protein (MBP) was found in Tfeb KO fish, and Tfeb overexpression reduced MBP mRNA levels in zebrafish central nervous system (CNS) (Meireles et al., 2018). The repressive role of TFEB also has been confirmed in a model of remyelinization after the injection of a demyelinating agent in the mouse brain (Meireles et al., 2018). Of note, unexpected higher levels of cytosol localized inactive TFEB were found in this model after injury (Meireles et al., 2018). These observations suggest an important role of MiT/TFE transcription factors in the regulation of myelinization in the CNS.

In summary, there is a progressive physiologic decline with aging, mainly affecting cognitive function, a process that may be exacerbated by external adverse conditions. As master regulators of cellular pathways involved in response to stress, MiT/TFE transcription factors are attractive targets for future studies aimed at the development of effective antiaging strategies. Given the functional conservation of these proteins across species, the use of different animal models would be fully warranted.

## Cell Proliferation, Differentiation, and Tumorigenesis

**FIGURE 9:**
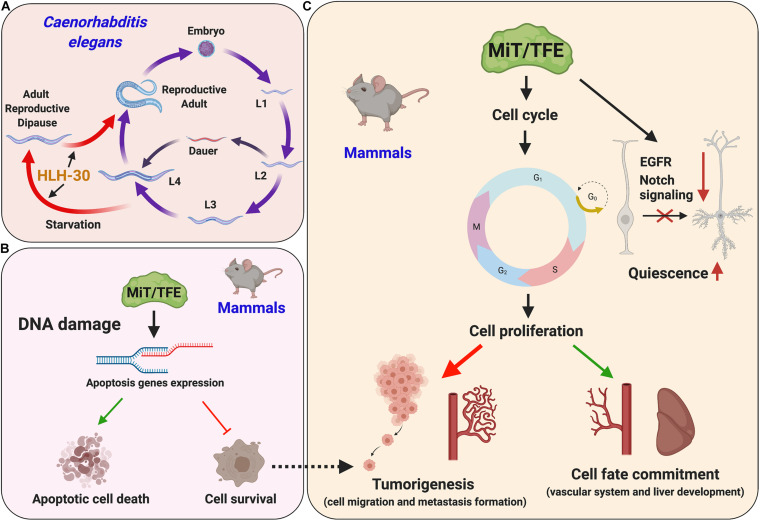
MiT/TFE transcription factors regulate cell fate and lineage decision. **(A)** In *C. elegans*, helix-loop-helix transcription factor 30 (HLH-30) regulates entry and recovery of adult reproductive quiescence state induced by starvation. **(B)** MiT/TFE transcription factors are activated under DNA damage, and depending on the severity of the damage, they can play a dual role inducing apoptosis and cell death or cell survival. **(C)** The MiT/TFE transcription factors regulate cell cycle and proliferation. TFEB can promote a quiescent state in neural stem cell by blocking pro-proliferative inputs. In addition, these transcription factors can promote proliferation rate in the liver and vascular system; however, they can also induce tumorigenesis.

Recent studies have shown that TFEB and TFE3 modulate the expression of genes involved in the cell cycle and apoptosis in response to genotoxic stress, suggesting that these transcription factors may trigger and coordinate cellular death pathways to prevent the spread of mutations to daughter cells (Brady et al., 2018a; Pisonero-Vaquero et al., 2020) (Figure 9 and Table 1). In particular, TFEB and TFE3 are activated by DNA-damaging agents, such as etoposide (Brady et al., 2018a) and doxorubicin (Pisonero-Vaquero et al., 2020), and participate in the DNA damage response through direct regulation, both at the transcriptional and protein levels of p53 and p21 proteins, known to be involved in cell cycle regulation and progression, and apoptosis. Of note, although TFEB and TFE3 seem to have a proapoptotic action upon treatment with etoposide (Brady et al., 2018a), the overexpression of TFEB in cancer cells in the presence or absence of genotoxic agents appears to promote cell survival (Pisonero-Vaquero et al., 2020). These findings suggest that the role of MiT/TFE transcription factors may vary depending on the nature of the DNA damage and cell context. Further investigation is required to better assess these observations.

The ability of MiT/TFE transcription factors, in particular TFEB, to regulate cell cycle and proliferation has recently been confirmed *in vivo* in a more physiological context. TFEB is involved in the regulation of the late phases of mouse embryonic development, particularly in G1-S cell cycle transition through the direct modulation of cyclin-dependent kinase (CDK)4 expression (Doronzo et al., 2019). Phosphorylation of the retinoblastoma protein by CDK4 leads to the release and activation of E2F, which in turn stimulates the expression of genes involved in the G1-S transition (Malumbres and Barbacid, 2009). Consequently, TFEB silencing in endothelial cells causes an accumulation of cells in G1 phase and a reduction of their proliferation rate (Doronzo et al., 2019). In addition, CDK4/6 interacts with and phosphorylates TFEB/TFE3 in the nucleus of cells in G1 phase promoting TFEB/TFE3 inactivation and export to the cytoplasm. Conversely, low CDK4/6 activity during S–M phases results in TFEB and TFE3 activation and induction of lysosomal biogenesis and autophagy (Yin et al., 2020).

Furthermore, TFEB has a profound impact on the fate of hepatoblasts, being responsible for the increased proliferation rate of liver bi-potent stem cells and their shift toward a cholangiocyte-like phenotype during embryogenesis or liver damage and repair. SRY-box transcription factor 9 (SOX9), which is transcriptionally regulated by TFEB, is implicated in this process; SOX9 overexpression upregulates the expression of biliary genes while repressing the hepatocyte pathways. It is important to note that the impairment of these biological processes may lead to cholangiocarcinoma (Pastore et al., 2020).

TFEB has also been shown to indirectly block cell proliferation by driving a more conventional transcriptional program, namely, regulation of lysosomal activity. Sustaining lysosomal proteolysis, TFEB controls the quiescent state of the neural stem cells in specific areas of adult mouse brain through the reduction in the levels of epidermal growth factor receptor and Notch signaling (Kobayashi et al., 2019). Therefore, pro-proliferative inputs are blocked, and neuronal stem cells maintain a quiescent state. In addition, the proportion of newly differentiated neurons to neuronal stem cells was significantly decreased in TFEB conditional KO mice, suggesting that TFEB may be required for neuronal differentiation (Kobayashi et al., 2019). Moreover, experiments performed in embryonic stem cells have demonstrated that TFEB has a role in endoderm specification, promoting the endo-lysosomal sequestration of GSK3β, thus allowing newly synthetized β-catenin to migrate to the nucleus. Thus, the expression of genes involved in tissue specification is regulated by the activation of TFEB through the AMPK-mediated inhibition of mTORC1 (Young et al., 2016). Then, perhaps not surprisingly, embryoid bodies generated from AMPK-depleted cells largely phenocopy aberrant tissue specifications observed in embryoid bodies derived from embryonic stem cells lacking TFEB. Furthermore, TFEB overexpression in AMPK KO embryonic stem cells largely rescues the developmental defects (Young et al., 2016).

Altogether, these findings underscore the importance of MiT/TFE transcription factors in regulating quiescent states and determining cell fate specification in both early and late development. In this regard, it is intriguing that HLH-30 has been recently defined as a master regulator of adult reproductive diapause in *C. elegans*, a state somewhat analogous to cell quiescence in that the condition is reversible and protective against adverse circumstances (Yao, 2014; Ahmad and Amiji, 2017; Gerisch et al., 2020).

The fine orchestration of cell cycle and cell differentiation is essential to avoid a non-physiological and excessive proliferation that may result in several pathological conditions including neoplastic disorders (Zhivotovsky and Orrenius, 2010). Indeed, several studies have linked MiT/TFE transcription factors to cancer. For instance, MITF gene is known to be involved in human melanomas, as indicated by the genetic amplification of MITF locus in most tumors and gene mutations in some (Goding and Arnheiter, 2019). A subset of pancreatic adenocarcinomas are characterized by a high expression of MITF, TFE3, and TFEB genes (Levy et al., 2006; Kauffman et al., 2014; Perera et al., 2015, 2019; Raben and Puertollano, 2016), whereas some renal cell carcinomas and alveolar soft sarcomas show chromosomal translocation of TFEB and TFE3 genes, leading to the formation of chimeric proteins or fusion of their coding regions with strong regulatory elements of unrelated genes resulting in the upregulation of the MiT/TFE gene network (Kuiper et al., 2003; Xie et al., 2019). These observations indicate that uncontrolled upregulation of the MiT/TFE target genes is linked to malignancy, but the molecular mechanisms underlying the oncogenic effects of the MiT/TFE proteins are far from being fully understood. This is particularly true in cases where a chromosomal translocation is involved (Raben and Puertollano, 2016; Perera et al., 2019).

It is known that the activation of autophagy provides energy to malignant cells. AMPK-dependent activation of TFE3 and TFEB was shown to sustain the proliferation and survival of murine lung adenocarcinoma cells in response to glucose deprivation (Eichner et al., 2019). These data fit nicely with the concept stipulating that malignant cells exploit autophagy to provide energy (Perera et al., 2015). In addition, the abovementioned role of MiT/TFE transcription factors in the cell cycle may also contribute to tumorigenesis. Likewise, the pro-proliferative activity of TFEB in endothelial cells, important for the formation of the vascular system during development, might drive tumor angiogenesis (Doronzo et al., 2019). Moreover, TFEB was shown to regulate quiescent states (Kobayashi et al., 2019; Gerisch et al., 2020), and several tumors rely on low-growing and stem-like cells to survive cancer treatments (Chen H. D. et al., 2017). In addition, a feedback loop has been proposed in which overexpression of TFEB, TFE3, or MITF upregulates RagD GTPase resulting in hyperactivation of mTORC1, induction of cellular proliferation, and cancer growth (Di Malta et al., 2017). Also, kidney-specific overexpression of TFEB in mice, which recapitulates the pathology observed in human kidney tumors, results in the dysregulation of the Wnt signaling pathway (Calcagni et al., 2016).

Metastases, the primary cause of cancer morbidity and mortality, are a common feature of different types of malignant tumors. They are the result of the migration of cancer cells from the original site of a primary tumor to colonize other tissues or organs (Seyfried and Huysentruyt, 2013). Interestingly, reduced migratory and invasive phenotype of lung cancer cell lines and oral squamous cell carcinomas have been associated with TFEB depletion (Giatromanolaki et al., 2015; Sakamoto et al., 2018), suggesting that the MiT/TFE proteins may regulate cellular migration.

The degradative activity of lysosomes may be required for metastatic processes. During lysosomal exocytosis, hydrolytic enzymes are released into the extracellular space where they digest extracellular matrix and disrupt cell–cell contact sites, delineating “anatomical routes” for the invading cancer cells (Kallunki et al., 2013; Davidson and Vander Heiden, 2017; Morgan et al., 2018). Recently, the lysosomal protein TMEM106B has been described as a metastasis-promoting factor (Kundu et al., 2018). TMEM106B pro-invasive effect is associated with TFEB activation and concomitant upregulation of lysosomal enzymes such as cathepsins. The accumulation of cathepsins inside the lysosomal lumen leads to Ca^2+^-mediated lysosomal exocytosis in lung cancer cells resulting in the release of proteases into the extracellular space. Treatment with cathepsin inhibitors is an efficient antitumoral therapeutic approach (Kundu et al., 2018), and Cathepsin D levels were shown to correlate with poor prognosis in non-small-cell lung cancer (Giatromanolaki et al., 2015). A pro-migration function of TFEB has also been reported in LPS-activated dendritic cells (Bretou et al., 2017; Irazoqui, 2020). Notably, dendritic cells may exert a dual role during tumorigenesis promoting either antitumor immune response or cancer growth (Wylie et al., 2019).

The dysregulation of these biological events may lead to malignant transformation and, as mentioned above, MiT/TFE genes have been linked to several tumors. A better characterization of the underlying molecular pathways is critical for the development of effective therapies. Mammals may be preferred over other models to monitor cell progression and growth. Nonetheless, tumorigenesis involves multiple steps, such as metabolic rearrangement and cell migration. Therefore, lower species such as *C. elegans* and *D. rerio*, where similar processes are controlled by MiT/TFE transcription factors, may be selected for their easy genetic manipulation and body transparency as well as for their utility in high-throughput drug screenings.

## Conclusion

The MiT/TFE family comprises a defined group of transcription factors that were discovered approximately three decades ago. However, the function of some of the members in the adaptation response to stress became evident only recently. The global transcriptional control of the MiT/TFE proteins in the organismal adaptation to stressful conditions positions them as prime master regulators. Indeed, how these transcription factors sense and integrate the distress signals to counter fasting, disruption of energy homeostasis, alteration in lipid and glucose metabolisms, pathogen infection, oxidative stress, mitochondrial dysfunction, aging-related conditions, and tumorigenesis is a subject of intense ongoing research.

Through the transcriptional regulation of the expression of hundreds of genes involved in different cellular pathways, the MiT/TFE transcription factors chiefly master the response to a variety of stressors and physiological challenges. These proteins could transcriptionally amplify pro-life genes promoting life span extension or lead to apoptosis and cell death under chronic stress.

Many of the critical regulatory roles ascribed to the MiT/TFE family are evolutionarily conserved. There is a remarkable basal mechanistic conservation across species down to lower metazoans, in which the presence of a single MiT/TFE transcription factor indicates that the adaptation to environmental stresses occurred early during evolution.

Studies in different animal models have undoubtedly contributed to the current understanding of the signaling pathways regulated by MiT/TFE proteins and demonstrated a significant functional conservation across species. Information gained from these studies will stimulate the development of new therapies for the treatment of several human diseases, including metabolic syndrome, lysosomal storage disorders, neurodegeneration, and cancer.

## Author Contributions

ML, PSC, AR, NKM, EJ, and JAM collected and analyzed the literature and shared in the writing. ML, PSC, AR, and JAM prepared the figures. JAM selected the subject material and wrote the manuscript. All authors contributed to the article and approved the submitted version.

## Conflict of Interest

The authors declare that the research was conducted in the absence of any commercial or financial relationships that could be construed as a potential conflict of interest.

## References

[B1] Ahmad G. Amiji M. M. (2017). Cancer stem cell-targeted therapeutics and delivery strategies. *Expert. Opin. Drug. Deliv.* 14 997–1008. 10.1080/17425247.2017.1263615 27866420

[B2] Awad O. Sarkar C. Panicker L. M. Miller D. Zeng X. Sgambato J. A. (2015). Altered TFEB-mediated lysosomal biogenesis in Gaucher disease iPSC-derived neuronal cells. *Hum. Mol. Genet.* 24 5775–5788. 10.1093/hmg/ddv297 26220978

[B3] Bahrami A. Bianconi V. Pirro M. Orafai H. M. Sahebkar A. (2020). The role of TFEB in tumor cell autophagy: diagnostic and therapeutic opportunities. *Life Sci.* 244:117341. 10.1016/j.lfs.2020.117341 31972208

[B4] Ballabio A. Bonifacino J. S. (2020). Lysosomes as dynamic regulators of cell and organismal homeostasis. *Nat. Rev. Mol. Cell Biol.* 21 101–118. 10.1038/s41580-019-0185-4 31768005

[B5] Bennett C. F. Kwon J. J. Chen C. Russell J. Acosta K. Burnaevskiy N. (2017). Transaldolase inhibition impairs mitochondrial respiration and induces a starvation-like longevity response in *Caenorhabditis elegans*. *PLoS Genet.* 13:e1006695. 10.1371/journal.pgen.1006695 28355222 PMC5389855

[B6] Betschinger J. Nichols J. Dietmann S. Corrin P. D. Paddison P. J. Smith A. (2013). Exit from pluripotency is gated by intracellular redistribution of the bHLH transcription factor Tfe3. *Cell* 153 335–347. 10.1016/j.cell.2013.03.012 23582324 PMC3661979

[B7] Bouche V. Espinosa A. P. Leone L. Sardiello M. Ballabio A. Botas J. (2016). Drosophila Mitf regulates the V-ATPase and the lysosomal-autophagic pathway. *Autophagy* 12 484–498. 10.1080/15548627.2015.1134081 26761346 PMC4835958

[B8] Brady O. A. Jeong E. Martina J. A. Pirooznia M. Tunc I. Puertollano R. (2018a). The transcription factors TFE3 and TFEB amplify p53 dependent transcriptional programs in response to DNA damage. *Elife* 7:e40856. 10.7554/eLife.40856 30520728 PMC6292694

[B9] Brady O. A. Martina J. A. Puertollano R. (2018b). Emerging roles for TFEB in the immune response and inflammation. *Autophagy* 14 181–189. 10.1080/15548627.2017.1313943 28738171 PMC5902167

[B10] Bretou M. Saez P. J. Sanseau D. Maurin M. Lankar D. Chabaud M. (2017). Lysosome signaling controls the migration of dendritic cells. *Sci. Immunol.* 2:eaak9573. 10.1126/sciimmunol.aak9573 29079589

[B11] Butler V. J. Gao F. Corrales C. I. Cortopassi W. A. Caballero B. Vohra M. (2019). Age- and stress-associated *C. elegans* granulins impair lysosomal function and induce a compensatory HLH-30/TFEB transcriptional response. *PLoS Genet.* 15:e1008295. 10.1371/journal.pgen.1008295 31398187 PMC6703691

[B12] Calcagni A. Kors L. Verschuren E. De Cegli R. Zampelli N. Nusco E. (2016). Modelling TFE renal cell carcinoma in mice reveals a critical role of WNT signaling. *Elife* 5:e17047. 10.7554/eLife.17047 27668431 PMC5036965

[B13] Campbell G. R. Rawat P. Bruckman R. S. Spector S. A. (2015). Human immunodeficiency virus type 1 Nef inhibits autophagy through transcription factor EB sequestration. *PLoS Pathog.* 11:e1005018. 10.1371/journal.ppat.1005018 26115100 PMC4482621

[B14] Chang K. T. Guo J. Di Ronza A. Sardiello M. (2018). Aminode: identification of evolutionary constraints in the human proteome. *Sci. Rep.* 8:1357. 10.1038/s41598-018-19744-w 29358731 PMC5778061

[B15] Chen H. D. Kao C. Y. Liu B. Y. Huang S. W. Kuo C. J. Ruan J. W. (2017). HLH-30/TFEB-mediated autophagy functions in a cell-autonomous manner for epithelium intrinsic cellular defense against bacterial pore-forming toxin in *C. elegans*. *Autophagy* 13 371–385. 10.1080/15548627.2016.1256933 27875098 PMC5324838

[B16] Chen L. Wang K. Long A. Jia L. Zhang Y. Deng H. (2017). Fasting-induced hormonal regulation of lysosomal function. *Cell Res.* 27 748–763. 10.1038/cr.2017.45 28374748 PMC5518872

[B17] Contreras P. S. Tapia P. J. Gonzalez-Hodar L. Peluso I. Soldati C. Napolitano G. (2020). c-Abl inhibition activates TFEB and promotes cellular clearance in a lysosomal disorder. *iScience* 23:101691. 10.1016/j.isci.2020.101691 33163944 PMC7607485

[B18] Cortes C. J. La Spada A. R. (2019). TFEB dysregulation as a driver of autophagy dysfunction in neurodegenerative disease: molecular mechanisms, cellular processes, and emerging therapeutic opportunities. *Neurobiol. Dis.* 122 83–93. 10.1016/j.nbd.2018.05.012 29852219 PMC6291370

[B19] Curran K. Lister J. A. Kunkel G. R. Prendergast A. Parichy D. M. Raible D. W. (2010). Interplay between Foxd3 and Mitf regulates cell fate plasticity in the zebrafish neural crest. *Dev. Biol.* 344 107–118. 10.1016/j.ydbio.2010.04.023 20460180 PMC2909359

[B20] Dall K. B. Faergeman N. J. (2019). Metabolic regulation of lifespan from a *C. elegans* perspective. *Genes Nutr.* 14:25. 10.1186/s12263-019-0650-x 31428207 PMC6694653

[B21] Davidson S. M. Vander Heiden M. G. (2017). Critical functions of the lysosome in cancer biology. *Annu. Rev. Pharmacol. Toxicol.* 57 481–507. 10.1146/annurev-pharmtox-010715-103101 27732799

[B22] Dhaunsi G. S. (2005). Molecular mechanisms of organelle biogenesis and related metabolic diseases. *Med. Princ. Pract.* 14(Suppl. 1) 49–57. 10.1159/000086184 16103713

[B23] Di Malta C. Siciliano D. Calcagni A. Monfregola J. Punzi S. Pastore N. (2017). Transcriptional activation of RagD GTPase controls mTORC1 and promotes cancer growth. *Science* 356 1188–1192. 10.1126/science.aag2553 28619945 PMC5730647

[B24] Diaz J. Berger S. Leon E. (2020). TFE3-associated neurodevelopmental disorder: a distinct recognizable syndrome. *Am. J. Med. Genet. A.* 182 584–590. 10.1002/ajmg.a.61437 31833172

[B25] Doronzo G. Astanina E. Cora D. Chiabotto G. Comunanza V. Noghero A. (2019). TFEB controls vascular development by regulating the proliferation of endothelial cells. *EMBO J.* 38:e98250 10.15252/embj.201798250 30591554 PMC6356157

[B26] Eichner L. J. Brun S. N. Herzig S. Young N. P. Curtis S. D. Shackelford D. B. (2019). Genetic analysis reveals AMPK is required to support tumor growth in murine kras-dependent lung cancer models. *Cell Metab.* 29 285.e287–302.e287. 10.1016/j.cmet.2018.10.005 30415923 PMC6365213

[B27] El Assar M. Angulo J. Rodriguez-Manas L. (2020). Frailty as a phenotypic manifestation of underlying oxidative stress. *Free Radic. Biol. Med.* 149 72–77. 10.1016/j.freeradbiomed.2019.08.011 31422077

[B28] El-Houjeiri L. Possik E. Vijayaraghavan T. Paquette M. Martina J. A. Kazan J. M. (2019). The transcription factors TFEB and TFE3 Link the FLCN-AMPK signaling axis to innate immune response and pathogen resistance. *Cell Rep.* 26 3613.e3613–3628.e3613. 10.1016/j.celrep.2019.02.102 30917316 PMC7457953

[B29] Ellis K. Bagwell J. Bagnat M. (2013). Notochord vacuoles are lysosome-related organelles that function in axis and spine morphogenesis. *J Cell. Biol.* 200 667–679. 10.1083/jcb.201212095 23460678 PMC3587825

[B30] Emanuel R. Sergin I. Bhattacharya S. Turner J. Epelman S. Settembre C. (2014). Induction of lysosomal biogenesis in atherosclerotic macrophages can rescue lipid-induced lysosomal dysfunction and downstream sequelae. *Arterioscler. Thromb. Vasc. Biol.* 34 1942–1952. 10.1161/ATVBAHA.114.303342 25060788 PMC4140993

[B31] Erlich A. T. Brownlee D. M. Beyfuss K. Hood D. A. (2018). Exercise induces TFEB expression and activity in skeletal muscle in a PGC-1alpha-dependent manner. *Am. J. Physiol. Cell Physiol.* 314 C62–C72. 10.1152/ajpcell.00162.2017 29046293 PMC5866381

[B32] Evans T. D. Zhang X. Jeong S. J. He A. Song E. Bhattacharya S. (2019). TFEB drives PGC-1alpha expression in adipocytes to protect against diet-induced metabolic dysfunction. *Sci. Signal.* 12:eaau2281. 10.1126/scisignal.aau2281 31690633 PMC6882500

[B33] Fernandez-Mosquera L. Diogo C. V. Yambire K. F. Santos G. L. Luna Sanchez M. Benit P. (2017). Acute and chronic mitochondrial respiratory chain deficiency differentially regulate lysosomal biogenesis. *Sci. Rep.* 7:45076. 10.1038/srep45076 28345620 PMC5366864

[B34] Franco-Juarez B. Mejia-Martinez F. Moreno-Arriola E. Hernandez-Vazquez A. Gomez-Manzo S. Marcial-Quino J. (2018). A high glucose diet induces autophagy in a HLH-30/TFEB-dependent manner and impairs the normal lifespan of *C. elegans*. *Aging* 10 2657–2667. 10.18632/aging.101577 30299269 PMC6224263

[B35] Fujimoto Y. Nakagawa Y. Satoh A. Okuda K. Shingyouchi A. Naka A. (2013). TFE3 controls lipid metabolism in adipose tissue of male mice by suppressing lipolysis and thermogenesis. *Endocrinology* 154 3577–3588. 10.1210/en.2013-1203 23885019

[B36] Gan L. Seki A. Shen K. Iyer H. Han K. Hayer A. (2019). The lysosomal GPCR-like protein GPR137B regulates rag and mTORC1 localization and activity. *Nat. Cell Biol.* 21 614–626. 10.1038/s41556-019-0321-6 31036939 PMC6649673

[B37] Gerisch B. Tharyan R. G. Mak J. Denzel S. I. Popkes-Van Oepen T. Henn N. (2020). HLH-30/tfeb is a master regulator of reproductive quiescence. *Dev. Cell* 53 316.e5–329.e5. 10.1016/j.devcel.2020.03.014 32302543

[B38] Ghanemi A. Yoshioka M. St-Amand J. (2018). Broken energy homeostasis and obesity pathogenesis: the surrounding concepts. *J. Clin. Med.* 7:453. 10.3390/jcm7110453 30463389 PMC6262529

[B39] Ghosh A. Jana M. Modi K. Gonzalez F. J. Sims K. B. Berry-Kravis E. (2015). Activation of peroxisome proliferator-activated receptor alpha induces lysosomal biogenesis in brain cells: implications for lysosomal storage disorders. *J. Biol. Chem.* 290 10309–10324. 10.1074/jbc.M114.610659 25750174 PMC4400343

[B40] Giatromanolaki A. Kalamida D. Sivridis E. Karagounis I. V. Gatter K. C. Harris A. L. (2015). Increased expression of transcription factor EB (TFEB) is associated with autophagy, migratory phenotype and poor prognosis in non-small cell lung cancer. *Lung Cancer* 90 98–105. 10.1016/j.lungcan.2015.07.008 26264650

[B41] Goding C. R. Arnheiter H. (2019). MITF-the first 25 years. *Genes Dev.* 33 983–1007. 10.1101/gad.324657.119 31123060 PMC6672050

[B42] Guerrero-Gomez D. Mora-Lorca J. A. Saenz-Narciso B. Naranjo-Galindo F. J. Munoz-Lobato F. Parrado-Fernandez C. (2019). Loss of glutathione redox homeostasis impairs proteostasis by inhibiting autophagy-dependent protein degradation. *Cell Death Differ.* 26 1545–1565. 10.1038/s41418-018-0270-9 30770874 PMC6748101

[B43] Gyoja F. (2014). A genome-wide survey of bHLH transcription factors in the Placozoan Trichoplax adhaerens reveals the ancient repertoire of this gene family in metazoan. *Gene* 542 29–37. 10.1016/j.gene.2014.03.024 24631262

[B44] Hallsson J. H. Haflidadottir B. S. Stivers C. Odenwald W. Arnheiter H. Pignoni F. (2004). The basic helix-loop-helix leucine zipper transcription factor Mitf is conserved in *Drosophila* and functions in eye development. *Genetics* 167 233–241. 10.1534/genetics.167.1.233 15166150 PMC1470875

[B45] Harvald E. B. Sprenger R. R. Dall K. B. Ejsing C. S. Nielsen R. Mandrup S. (2017). Multi-omics analyses of starvation responses reveal a central role for lipoprotein metabolism in acute starvation survival in *C. elegans*. *Cell Syst.* 5 38.e34–52.e34. 10.1016/j.cels.2017.06.004 28734827

[B46] Hemesath T. J. Steingrimsson E. Mcgill G. Hansen M. J. Vaught J. Hodgkinson C. A. (1994). microphthalmia, a critical factor in melanocyte development, defines a discrete transcription factor family. *Genes Dev.* 8 2770–2780. 10.1101/gad.8.22.2770 7958932

[B47] Hsu C. L. Lee E. X. Gordon K. L. Paz E. A. Shen W. C. Ohnishi K. (2018). MAP4K3 mediates amino acid-dependent regulation of autophagy via phosphorylation of TFEB. *Nat. Commun.* 9:942. 10.1038/s41467-018-03340-7 29507340 PMC5838220

[B48] Hu Z. Li H. Jiang H. Ren Y. Yu X. Qiu J. (2020). Transient inhibition of mTOR in human pluripotent stem cells enables robust formation of mouse-human chimeric embryos. *Sci. Adv.* 6:eaaz0298 10.1126/sciadv.aaz0298 PMC7220352 32426495

[B49] Huber K. Hofer D. C. Trefely S. Pelzmann H. J. Madreiter-Sokolowski C. Duta-Mare M. (2019). N-acetylaspartate pathway is nutrient responsive and coordinates lipid and energy metabolism in brown adipocytes. *Biochim. Biophys. Acta Mol. Cell Res.* 1866 337–348. 10.1016/j.bbamcr.2018.08.017 30595160 PMC6390944

[B50] Irazoqui J. E. (2020). Key Roles of MiT transcription factors in innate immunity and inflammation. *Trends Immunol.* 41 157–171. 10.1016/j.it.2019.12.003 31959514 PMC6995440

[B51] Iwasaki H. Naka A. Iida K. T. Nakagawa Y. Matsuzaka T. Ishii K. A. (2012). TFE3 regulates muscle metabolic gene expression, increases glycogen stores, and enhances insulin sensitivity in mice. *Am. J. Physiol. Endocrinol. Metab.* 302 E896–E902. 10.1152/ajpendo.00204.2011 22297304

[B52] Jones D. T. Taylor W. R. Thornton J. M. (1992). The rapid generation of mutation data matrices from protein sequences. *Comput. Appl. Biosci.* 8 275–282. 10.1093/bioinformatics/8.3.275 1633570

[B53] Kahn C. R. Wang G. Lee K. Y. (2019). Altered adipose tissue and adipocyte function in the pathogenesis of metabolic syndrome. *J. Clin. Invest.* 129 3990–4000. 10.1172/JCI129187 31573548 PMC6763230

[B54] Kallunki T. Olsen O. D. Jaattela M. (2013). Cancer-associated lysosomal changes: friends or foes? *Oncogene* 32 1995–2004. 10.1038/onc.2012.292 22777359

[B55] Kauffman E. C. Ricketts C. J. Rais-Bahrami S. Yang Y. Merino M. J. Bottaro D. P. (2014). Molecular genetics and cellular features of TFE3 and TFEB fusion kidney cancers. *Nat. Rev. Urol.* 11 465–475. 10.1038/nrurol.2014.162 25048860 PMC4551450

[B56] Kawakami A. Fisher D. E. (2017). The master role of microphthalmia-associated transcription factor in melanocyte and melanoma biology. *Lab. Invest.* 97 649–656. 10.1038/labinvest.2017.9 28263292

[B57] Kenyon C. (2010). A pathway that links reproductive status to lifespan in *Caenorhabditis elegans*. *Ann. N. Y. Acad. Sci.* 1204 156–162. 10.1111/j.1749-6632.2010.05640.x 20738286

[B58] Kharitonenkov A. Shiyanova T. L. Koester A. Ford A. M. Micanovic R. Galbreath E. J. (2005). FGF-21 as a novel metabolic regulator. *J. Clin. Invest.* 115 1627–1635. 10.1172/JCI23606 15902306 PMC1088017

[B59] Kim D. K. Lim H. S. Kawasaki I. Shim Y. H. Vaikath N. N. El-Agnaf O. M. (2016). Anti-aging treatments slow propagation of synucleinopathy by restoring lysosomal function. *Autophagy* 12 1849–1863. 10.1080/15548627.2016.1207014 27485532 PMC5079673

[B60] Kim H. J. Joe Y. Rah S. Y. Kim S. K. Park S. U. Park J. (2018). Carbon monoxide-induced TFEB nuclear translocation enhances mitophagy/mitochondrial biogenesis in hepatocytes and ameliorates inflammatory liver injury. *Cell Death. Dis.* 9:1060. 10.1038/s41419-018-1112-x 30333475 PMC6193007

[B61] Kinghorn K. J. Gronke S. Castillo-Quan J. I. Woodling N. S. Li L. Sirka E. (2016). A *Drosophila* model of neuronopathic gaucher disease demonstrates lysosomal-autophagic defects and altered mTOR signalling and is functionally rescued by rapamycin. *J. Neurosci.* 36 11654–11670. 10.1523/JNEUROSCI.4527-15.2016 27852774 PMC5125225

[B62] Kobayashi T. Piao W. Takamura T. Kori H. Miyachi H. Kitano S. (2019). Enhanced lysosomal degradation maintains the quiescent state of neural stem cells. *Nat. Commun.* 10:5446. 10.1038/s41467-019-13203-4 31784517 PMC6884460

[B63] Kuiper R. P. Schepens M. Thijssen J. Van Asseldonk M. Van Den Berg E. Bridge J. (2003). Upregulation of the transcription factor TFEB in t(6;11)(p21;q13)-positive renal cell carcinomas due to promoter substitution. *Hum. Mol. Genet.* 12 1661–1669. 10.1093/hmg/ddg178 12837690

[B64] Kumar S. Stecher G. Li M. Knyaz C. Tamura K. (2018). MEGA X: molecular evolutionary genetics analysis across computing platforms. *Mol. Biol. Evol.* 35 1547–1549. 10.1093/molbev/msy096 29722887 PMC5967553

[B65] Kumsta C. Chang J. T. Schmalz J. Hansen M. (2017). Hormetic heat stress and HSF-1 induce autophagy to improve survival and proteostasis in *C. elegans*. *Nat. Commun.* 8:14337. 10.1038/ncomms14337 28198373 PMC5316864

[B66] Kundu S. T. Grzeskowiak C. L. Fradette J. J. Gibson L. A. Rodriguez L. B. Creighton C. J. (2018). TMEM106B drives lung cancer metastasis by inducing TFEB-dependent lysosome synthesis and secretion of cathepsins. *Nat. Commun.* 9:2731. 10.1038/s41467-018-05013-x 30013069 PMC6048095

[B67] Kuo C. J. Hansen M. Troemel E. (2018). Autophagy and innate immunity: insights from invertebrate model organisms. *Autophagy* 14 233–242. 10.1080/15548627.2017.1389824 29130360 PMC5902216

[B68] Labbadia J. Brielmann R. M. Neto M. F. Lin Y. F. Haynes C. M. Morimoto R. I. (2017). Mitochondrial stress restores the heat shock response and prevents proteostasis collapse during aging. *Cell Rep.* 21 1481–1494. 10.1016/j.celrep.2017.10.038 29117555 PMC5726777

[B69] Lapierre L. R. De Magalhaes Filho C. D. Mcquary P. R. Chu C. C. Visvikis O. Chang J. T. (2013). The TFEB orthologue HLH-30 regulates autophagy and modulates longevity in Caenorhabditis elegans. *Nat. Commun.* 4:2267. 10.1038/ncomms3267 23925298 PMC3866206

[B70] Leclerc J. Garandeau D. Pandiani C. Gaudel C. Bille K. Nottet N. (2019). Lysosomal acid ceramidase ASAH1 controls the transition between invasive and proliferative phenotype in melanoma cells. *Oncogene* 38 1282–1295. 10.1038/s41388-018-0500-0 30254208

[B71] Ledent V. Vervoort M. (2001). The basic helix-loop-helix protein family: comparative genomics and phylogenetic analysis. *Genome Res.* 11 754–770. 10.1101/gr.177001 11337472 PMC311049

[B72] Lee K. Mylonakis E. (2017). An intestine-derived neuropeptide controls avoidance behavior in *Caenorhabditis elegans*. *Cell Rep.* 20 2501–2512. 10.1016/j.celrep.2017.08.053 28877481

[B73] Leiser S. F. Miller H. Rossner R. Fletcher M. Leonard A. Primitivo M. (2015). Cell nonautonomous activation of flavin-containing monooxygenase promotes longevity and health span. *Science* 350 1375–1378. 10.1126/science.aac9257 26586189 PMC4801033

[B74] Leow S. M. Chua S. X. Venkatachalam G. Shen L. Luo L. Clement M. V. (2017). Sub-lethal oxidative stress induces lysosome biogenesis via a lysosomal membrane permeabilization-cathepsin-caspase 3-transcription factor EB-dependent pathway. *Oncotarget* 8 16170–16189. 10.18632/oncotarget.14016 28002813 PMC5369955

[B75] Levy C. Khaled M. Fisher D. E. (2006). MITF: master regulator of melanocyte development and melanoma oncogene. *Trends Mol. Med.* 12 406–414. 10.1016/j.molmed.2006.07.008 16899407

[B76] Li J. Wada S. Weaver L. K. Biswas C. Behrens E. M. Arany Z. (2019). Myeloid Folliculin balances mTOR activation to maintain innate immunity homeostasis. *JCI Insight.* 5:e126939. 10.1172/jci.insight.126939 30843872 PMC6483010

[B77] Li Y. Xu M. Ding X. Yan C. Song Z. Chen L. (2016). Protein kinase C controls lysosome biogenesis independently of mTORC1. *Nat. Cell Biol.* 18 1065–1077. 10.1038/ncb3407 27617930

[B78] Lim J. Yue Z. (2015). Neuronal aggregates: formation, clearance, and spreading. *Dev. Cell* 32 491–501. 10.1016/j.devcel.2015.02.002 25710535 PMC4376477

[B79] Lin X. X. Sen I. Janssens G. E. Zhou X. Fonslow B. R. Edgar D. (2018). DAF-16/FOXO and HLH-30/TFEB function as combinatorial transcription factors to promote stress resistance and longevity. *Nat. Commun.* 9:4400. 10.1038/s41467-018-06624-0 30353013 PMC6199276

[B80] Lister J. A. Lane B. M. Nguyen A. Lunney K. (2011). Embryonic expression of zebrafish MiT family genes tfe3b, tfeb, and tfec. *Dev. Dyn.* 240 2529–2538. 10.1002/dvdy.22743 21932325 PMC3197887

[B81] Liu Y. Xin Y. Ye F. Wang W. Lu Q. Kaplan H. J. (2010). Taz-tead1 links cell-cell contact to zeb1 expression, proliferation, and dedifferentiation in retinal pigment epithelial cells. *Invest. Ophthalmol. Vis. Sci.* 51 3372–3378. 10.1167/iovs.09-4321 20207963 PMC2904003

[B82] Liu Y. J. Mcintyre R. L. Janssens G. E. Williams E. G. Lan J. Van Weeghel M. (2020). Mitochondrial translation and dynamics synergistically extend lifespan in *C. elegans* through HLH-30. *J. Cell Biol.* 219:e201907067 10.1083/jcb.201907067 PMC7265311 32259199

[B83] Malumbres M. Barbacid M. (2009). Cell cycle, CDKs and cancer: a changing paradigm. *Nat. Rev. Cancer* 9 153–166. 10.1038/nrc2602 19238148

[B84] Mansueto G. Armani A. Viscomi C. D’orsi L. De Cegli R. Polishchuk E. V. (2017). Transcription factor EB controls metabolic flexibility during exercise. *Cell Metab.* 25 182–196. 10.1016/j.cmet.2016.11.003 28011087 PMC5241227

[B85] Martina J. A. Chen Y. Gucek M. Puertollano R. (2012). MTORC1 functions as a transcriptional regulator of autophagy by preventing nuclear transport of TFEB. *Autophagy* 8 903–914. 10.4161/auto.19653 22576015 PMC3427256

[B86] Martina J. A. Diab H. I. Brady O. A. Puertollano R. (2016). TFEB and TFE3 are novel components of the integrated stress response. *EMBO J.* 35 479–495. 10.15252/embj.201593428 26813791 PMC4772850

[B87] Martina J. A. Diab H. I. Li H. Puertollano R. (2014a). Novel roles for the MiTF/TFE family of transcription factors in organelle biogenesis, nutrient sensing, and energy homeostasis. *Cell Mol. Life Sci.* 71 2483–2497. 10.1007/s00018-014-1565-8 24477476 PMC4057939

[B88] Martina J. A. Diab H. I. Lishu L. Jeong A. L. Patange S. Raben N. (2014b). The nutrient-responsive transcription factor TFE3 promotes autophagy, lysosomal biogenesis, and clearance of cellular debris. *Sci. Signal* 7:ra9. 10.1126/scisignal.2004754 24448649 PMC4696865

[B89] Martina J. A. Puertollano R. (2013). Rag GTPases mediate amino acid-dependent recruitment of TFEB and MITF to lysosomes. *J. Cell Biol.* 200 475–491. 10.1083/jcb.201209135 23401004 PMC3575543

[B90] Martina J. A. Puertollano R. (2018). Protein phosphatase 2A stimulates activation of TFEB and TFE3 transcription factors in response to oxidative stress. *J. Biol. Chem.* 293 12525–12534. 10.1074/jbc.RA118.003471 29945972 PMC6093222

[B91] Medina D. L. Di Paola S. Peluso I. Armani A. De Stefani D. Venditti R. (2015). Lysosomal calcium signalling regulates autophagy through calcineurin and TFEB. *Nat. Cell Biol.* 17 288–299. 10.1038/ncb3114 25720963 PMC4801004

[B92] Medina D. L. Fraldi A. Bouche V. Annunziata F. Mansueto G. Spampanato C. (2011). Transcriptional activation of lysosomal exocytosis promotes cellular clearance. *Dev. Cell* 21 421–430. 10.1016/j.devcel.2011.07.016 21889421 PMC3173716

[B93] Meireles A. M. Shen K. Zoupi L. Iyer H. Bouchard E. L. Williams A. (2018). The lysosomal transcription factor tfeb represses myelination downstream of the rag-ragulator complex. *Dev. Cell* 47:e315. 10.1016/j.devcel.2018.10.003 PMC6250074 30399334

[B94] Miller A. J. Levy C. Davis I. J. Razin E. Fisher D. E. (2005). Sumoylation of MITF and its related family members TFE3 and TFEB. *J. Biol. Chem.* 280 146–155. 10.1074/jbc.M411757200 15507434

[B95] Moller K. Sigurbjornsdottir S. Arnthorsson A. O. Pogenberg V. Dilshat R. Fock V. (2019). MITF has a central role in regulating starvation-induced autophagy in melanoma. *Sci. Rep.* 9:1055. 10.1038/s41598-018-37522-6 30705290 PMC6355916

[B96] Morgan M. J. Fitzwalter B. E. Owens C. R. Powers R. K. Sottnik J. L. Gamez G. (2018). Metastatic cells are preferentially vulnerable to lysosomal inhibition. *Proc. Natl. Acad. Sci. U.S.A.* 115 E8479–E8488. 10.1073/pnas.1706526115 30127018 PMC6130375

[B97] Mullock B. Luzio J. (2005). “Theory of organelle biogenesis: a historical perspective,” in *The Biogenesis of Cellular Organelles*, ed. Mullins C. (Boston, MA: Springer), 1–18. 10.1007/0-387-26867-7_1

[B98] Murphy J. T. Liu H. Ma X. Shaver A. Egan B. M. Oh C. (2019). Simple nutrients bypass the requirement for HLH-30 in coupling lysosomal nutrient sensing to survival. *PLoS Biol.* 17:e3000245. 10.1371/journal.pbio.3000245 31086360 PMC6516633

[B99] Nabar N. R. Kehrl J. H. (2017). The transcription factor EB links cellular stress to the immune response. *Yale J. Biol. Med.* 90 301–315. 28656016 PMC5482306

[B100] Najibi M. Labed S. A. Visvikis O. Irazoqui J. E. (2016). An evolutionarily conserved PLC-PKD-TFEB Pathway for host defense. *Cell Rep.* 15 1728–1742. 10.1016/j.celrep.2016.04.052 27184844 PMC4880541

[B101] Nakagawa Y. Shimano H. Yoshikawa T. Ide T. Tamura M. Furusawa M. (2006). TFE3 transcriptionally activates hepatic IRS-2, participates in insulin signaling and ameliorates diabetes. *Nat. Med.* 12 107–113. 10.1038/nm1334 16327801

[B102] Nakamura S. Karalay O. Jager P. S. Horikawa M. Klein C. Nakamura K. (2016). Mondo complexes regulate TFEB via TOR inhibition to promote longevity in response to gonadal signals. *Nat. Commun.* 7:10944. 10.1038/ncomms10944 27001890 PMC4804169

[B103] Napolitano G. Di Malta C. Esposito A. De Araujo M. E. G. Pece S. Bertalot G. (2020). A substrate-specific mTORC1 pathway underlies Birt-Hogg-Dube syndrome. *Nature* 585 597–602. 10.1038/s41586-020-2444-0 32612235 PMC7610377

[B104] Napolitano G. Esposito A. Choi H. Matarese M. Benedetti V. Di Malta C. (2018). mTOR-dependent phosphorylation controls TFEB nuclear export. *Nat. Commun.* 9:3312. 10.1038/s41467-018-05862-6 30120233 PMC6098152

[B105] Nezich C. L. Wang C. Fogel A. I. Youle R. J. (2015). MiT/TFE transcription factors are activated during mitophagy downstream of Parkin and Atg5. *J. Cell Biol.* 210 435–450. 10.1083/jcb.201501002 26240184 PMC4523611

[B106] O’Rourke E. J. Ruvkun G. (2013). MXL-3 and HLH-30 transcriptionally link lipolysis and autophagy to nutrient availability. *Nat. Cell Biol.* 15 668–676. 10.1038/ncb2741 23604316 PMC3723461

[B107] Ouimet M. Koster S. Sakowski E. Ramkhelawon B. Van Solingen C. Oldebeken S. (2016). Mycobacterium tuberculosis induces the miR-33 locus to reprogram autophagy and host lipid metabolism. *Nat. Immunol.* 17 677–686. 10.1038/ni.3434 27089382 PMC4873392

[B108] Palikaras K. Mari M. Petanidou B. Pasparaki A. Filippidis G. Tavernarakis N. (2017). Ectopic fat deposition contributes to age-associated pathology in *Caenorhabditis elegans*. *J. Lipid Res.* 58 72–80. 10.1194/jlr.M069385 27884963 PMC5234711

[B109] Palmieri M. Impey S. Kang H. Di Ronza A. Pelz C. Sardiello M. (2011). Characterization of the CLEAR network reveals an integrated control of cellular clearance pathways. *Hum. Mol. Genet.* 20 3852–3866. 10.1093/hmg/ddr306 21752829

[B110] Palmieri M. Pal R. Nelvagal H. R. Lotfi P. Stinnett G. R. Seymour M. L. (2017). mTORC1-independent TFEB activation via Akt inhibition promotes cellular clearance in neurodegenerative storage diseases. *Nat. Commun.* 8:14338. 10.1038/ncomms14338 28165011 PMC5303831

[B111] Pastore N. Brady O. A. Diab H. I. Martina J. A. Sun L. Huynh T. (2016). TFEB and TFE3 cooperate in the regulation of the innate immune response in activated macrophages. *Autophagy* 12 1240–1258. 10.1080/15548627.2016.1179405 27171064 PMC4968228

[B112] Pastore N. Huynh T. Herz N. J. Calcagni A. Klisch T. J. Brunetti L. (2020). TFEB regulates murine liver cell fate during development and regeneration. *Nat. Commun.* 11:2461 10.1038/s41467-020-16300-x PMC7235048 32424153

[B113] Pastore N. Vainshtein A. Klisch T. J. Armani A. Huynh T. Herz N. J. (2017). TFE3 regulates whole-body energy metabolism in cooperation with TFEB. *EMBO Mol. Med.* 9 605–621. 10.15252/emmm.201607204 28283651 PMC5412821

[B114] Perera R. M. Di Malta C. Ballabio A. (2019). MiT/TFE family of transcription factors, lysosomes, and cancer. *Annu. Rev. Cancer Biol.* 3 203–222. 10.1146/annurev-cancerbio-030518-055835 31650096 PMC6812561

[B115] Perera R. M. Stoykova S. Nicolay B. N. Ross K. N. Fitamant J. Boukhali M. (2015). Transcriptional control of autophagy-lysosome function drives pancreatic cancer metabolism. *Nature* 524 361–365. 10.1038/nature14587 26168401 PMC5086585

[B116] Pisonero-Vaquero S. Soldati C. Cesana M. Ballabio A. Medina D. L. (2020). TFEB modulates p21/WAF1/CIP1 during the DNA damage response. *Cells* 9:1186 10.3390/cells9051186 PMC7290768 32397616

[B117] Ploper D. Taelman V. F. Robert L. Perez B. S. Titz B. Chen H. W. (2015). MITF drives endolysosomal biogenesis and potentiates Wnt signaling in melanoma cells. *Proc. Natl. Acad. Sci. U.S.A.* 112 E420–E429. 10.1073/pnas.1424576112 25605940 PMC4321275

[B118] Pogenberg V. Ballesteros-Alvarez J. Schober R. Sigvaldadottir I. Obarska-Kosinska A. Milewski M. (2020). Mechanism of conditional partner selectivity in MITF/TFE family transcription factors with a conserved coiled coil stammer motif. *Nucleic Acids Res.* 48 934–948. 10.1093/nar/gkz1104 31777941 PMC6954422

[B119] Popov L. D. (2020). Mitochondrial biogenesis: an update. *J. Cell Mol. Med.* 24 4892–4899. 10.1111/jcmm.15194 32279443 PMC7205802

[B120] Puertollano R. Ferguson S. M. Brugarolas J. Ballabio A. (2018). The complex relationship between TFEB transcription factor phosphorylation and subcellular localization. *EMBO J.* 37:e98804. 10.15252/embj.201798804 29764979 PMC5983138

[B121] Raben N. Puertollano R. (2016). TFEB and TFE3: linking lysosomes to cellular adaptation to stress. *Annu. Rev. Cell Dev. Biol.* 32 255–278. 10.1146/annurev-cellbio-111315-125407 27298091 PMC6490169

[B122] Rehli M. Den Elzen N. Cassady A. I. Ostrowski M. C. Hume D. A. (1999). Cloning and characterization of the murine genes for bHLH-ZIP transcription factors TFEC and TFEB reveal a common gene organization for all MiT subfamily members. *Genomics* 56 111–120. 10.1006/geno.1998.5588 10036191

[B123] Roczniak-Ferguson A. Petit C. S. Froehlich F. Qian S. Ky J. Angarola B. (2012). The transcription factor TFEB links mTORC1 signaling to transcriptional control of lysosome homeostasis. *Sci. Signal.* 5:ra42. 10.1126/scisignal.2002790 22692423 PMC3437338

[B124] Sakamoto H. Yamashita K. Okamoto K. Kadowaki T. Sakai E. Umeda M. (2018). Transcription factor EB influences invasion and migration in oral squamous cell carcinomas. *Oral Dis.* 24 741–748. 10.1111/odi.12826 29316035

[B125] Salma N. Song J. S. Arany Z. Fisher D. E. (2015). Transcription factor Tfe3 directly regulates Pgc-1alpha in muscle. *J. Cell Physiol.* 230 2330–2336. 10.1002/jcp.24978 25736533 PMC4617629

[B126] Sardiello M. Ballabio A. (2009). Lysosomal enhancement: a CLEAR answer to cellular degradative needs. *Cell Cycle* 8 4021–4022. 10.4161/cc.8.24.10263 19949301

[B127] Sardiello M. Palmieri M. Di Ronza A. Medina D. L. Valenza M. Gennarino V. A. (2009). A gene network regulating lysosomal biogenesis and function. *Science* 325 473–477. 10.1126/science.1174447 19556463

[B128] Settembre C. De Cegli R. Mansueto G. Saha P. K. Vetrini F. Visvikis O. (2013). TFEB controls cellular lipid metabolism through a starvation-induced autoregulatory loop. *Nat. Cell Biol.* 15 647–658. 10.1038/ncb2718 23604321 PMC3699877

[B129] Settembre C. Di Malta C. Polito V. A. Garcia Arencibia M. Vetrini F. Erdin S. (2011). TFEB links autophagy to lysosomal biogenesis. *Science* 332 1429–1433. 10.1126/science.1204592 21617040 PMC3638014

[B130] Settembre C. Zoncu R. Medina D. L. Vetrini F. Erdin S. Erdin S. (2012). A lysosome-to-nucleus signalling mechanism senses and regulates the lysosome via mTOR and TFEB. *EMBO J.* 31 1095–1108. 10.1038/emboj.2012.32 22343943 PMC3298007

[B131] Seyfried T. N. Huysentruyt L. C. (2013). On the origin of cancer metastasis. *Crit. Rev. Oncog.* 18 43–73. 10.1615/critrevoncog.v18.i1-2.40 23237552 PMC3597235

[B132] Sha Y. Rao L. Settembre C. Ballabio A. Eissa N. T. (2017). STUB1 regulates TFEB-induced autophagy-lysosome pathway. *EMBO J.* 36 2544–2552. 10.15252/embj.201796699 28754656 PMC5579343

[B133] Silvestrini M. J. Johnson J. R. Kumar A. V. Thakurta T. G. Blais K. Neill Z. A. (2018). nuclear export inhibition enhances HLH-30/TFEB activity, autophagy, and lifespan. *Cell Rep.* 23 1915–1921. 29768192 10.1016/j.celrep.2018.04.063 PMC5991088

[B134] Simionato E. Ledent V. Richards G. Thomas-Chollier M. Kerner P. Coornaert D. (2007). Origin and diversification of the basic helix-loop-helix gene family in metazoans: insights from comparative genomics. *BMC Evol. Biol.* 7:33. 10.1186/1471-2148-7-33 17335570 PMC1828162

[B135] Singh N. Kansal P. Ahmad Z. Baid N. Kushwaha H. Khatri N. (2018). Antimycobacterial effect of IFNG (interferon gamma)-induced autophagy depends on HMOX1 (heme oxygenase 1)-mediated increase in intracellular calcium levels and modulation of PPP3/calcineurin-TFEB (transcription factor EB) axis. *Autophagy* 14 972–991. 10.1080/15548627.2018.1436936 29457983 PMC6103408

[B136] Slade L. Pulinilkunnil T. (2017). The MiTF/TFE family of transcription factors: master regulators of organelle signaling, metabolism, and stress adaptation. *Mol. Cancer Res.* 15 1637–1643. 10.1158/1541-7786.MCR-17-0320 28851811

[B137] Spampanato C. Feeney E. Li L. Cardone M. Lim J. A. Annunziata F. (2013). Transcription factor EB (TFEB) is a new therapeutic target for Pompe disease. *EMBO Mol. Med.* 5 691–706. 10.1002/emmm.201202176 23606558 PMC3662313

[B138] Steingrimsson E. Copeland N. G. Jenkins N. A. (2004). Melanocytes and the microphthalmia transcription factor network. *Annu. Rev. Genet.* 38 365–411. 10.1146/annurev.genet.38.072902.092717 15568981

[B139] Steingrimsson E. Tessarollo L. Reid S. W. Jenkins N. A. Copeland N. G. (1998). The bHLH-Zip transcription factor Tfeb is essential for placental vascularization. *Development* 125 4607–4616. 9806910 10.1242/dev.125.23.4607

[B140] Sun X. Zhou Y. Zhang R. Wang Z. Xu M. Zhang D. (2020). Dstyk mutation leads to congenital scoliosis-like vertebral malformations in zebrafish via dysregulated mTORC1/TFEB pathway. *Nat. Commun.* 11:479. 10.1038/s41467-019-14169-z 31980602 PMC6981171

[B141] Taylor J. S. Van De Peer Y. Meyer A. (2001). Revisiting recent challenges to the ancient fish-specific genome duplication hypothesis. *Curr. Biol.* 11 R1005–R1008. 10.1016/s0960-9822(01)00610-8 11747834

[B142] Tognon E. Kobia F. Busi I. Fumagalli A. De Masi F. Vaccari T. (2016). Control of lysosomal biogenesis and Notch-dependent tissue patterning by components of the TFEB-V-ATPase axis in Drosophila melanogaster. *Autophagy* 12 499–514. 10.1080/15548627.2015.1134080 26727288 PMC4836007

[B143] Villegas F. Lehalle D. Mayer D. Rittirsch M. Stadler M. B. Zinner M. (2019). Lysosomal signaling licenses embryonic stem cell differentiation via inactivation of Tfe3. *Cell Stem Cell.* 24 257.e258–270.e258. 10.1016/j.stem.2018.11.021 30595499

[B144] Visvikis O. Ihuegbu N. Labed S. A. Luhachack L. G. Alves A. F. Wollenberg A. C. (2014). Innate host defense requires TFEB-mediated transcription of cytoprotective and antimicrobial genes. *Immunity* 40 896–909. 10.1016/j.immuni.2014.05.002 24882217 PMC4104614

[B145] Wada S. Neinast M. Jang C. Ibrahim Y. H. Lee G. Babu A. (2016). The tumor suppressor FLCN mediates an alternate mTOR pathway to regulate browning of adipose tissue. *Genes Dev.* 30 2551–2564. 10.1101/gad.287953.116 27913603 PMC5159669

[B146] Wang H. Wang N. Xu D. Ma Q. Chen Y. Xu S. (2019). Oxidation of multiple MiT/TFE transcription factors links oxidative stress to transcriptional control of autophagy and lysosome biogenesis. *Autophagy* 16 1683–1696. 10.1080/15548627.2019.1704104 31826695 PMC8386635

[B147] Wang S. Chen Y. Li X. Zhang W. Liu Z. Wu M. (2020). Emerging role of transcription factor EB in mitochondrial quality control. *Biomed. Pharmacother.* 128:110272. 10.1016/j.biopha.2020.110272 32447212

[B148] Wang Y. Huang Y. Liu J. Zhang J. Xu M. You Z. (2020). Acetyltransferase GCN5 regulates autophagy and lysosome biogenesis by targeting TFEB. *EMBO Rep.* 21:e48335. 10.15252/embr.201948335 31750630 PMC6945067

[B149] Wu M. Gibbons J. G. Deloid G. M. Bedugnis A. S. Thimmulappa R. K. Biswal S. (2017). Immunomodulators targeting MARCO expression improve resistance to postinfluenza bacterial pneumonia. *Am. J. Physiol. Lung Cell Mol. Physiol.* 313 L138–L153. 10.1152/ajplung.00075.2017 28408365 PMC5538876

[B150] Wylie B. Macri C. Mintern J. D. Waithman J. (2019). Dendritic cells and cancer: from biology to therapeutic intervention. *Cancers* 11:521. 10.3390/cancers11040521 30979057 PMC6521027

[B151] Xie L. Zhang Y. Wu C. L. (2019). Microphthalmia family of transcription factors associated renal cell carcinoma. *Asian J. Urol.* 6 312–320. 10.1016/j.ajur.2019.04.003 31768316 PMC6872788

[B152] Yang C. Chen Y. Li Z. Cao H. Chen K. Lai P. (2017). Chondrocyte-specific knockout of TSC-1 leads to congenital spinal deformity in mice. *Biomed. Res. Int.* 2017:8215805. 10.1155/2017/8215805 28523278 PMC5420956

[B153] Yang M. Liu E. Tang L. Lei Y. Sun X. Hu J. (2018). Emerging roles and regulation of MiT/TFE transcriptional factors. *Cell Commun. Signal.* 16:31. 10.1186/s12964-018-0242-1 29903018 PMC6003119

[B154] Yao G. (2014). Modelling mammalian cellular quiescence. *Interface Focus* 4:20130074. 10.1098/rsfs.2013.0074 24904737 PMC3996586

[B155] Yin Q. Jian Y. Xu M. Huang X. Wang N. Liu Z. (2020). CDK4/6 regulate lysosome biogenesis through TFEB/TFE3. *J. Cell Biol.* 219:e201911036. 10.1083/jcb.201911036 32662822 PMC7401801

[B156] Young N. P. Kamireddy A. Van Nostrand J. L. Eichner L. J. Shokhirev M. N. Dayn Y. (2016). AMPK governs lineage specification through Tfeb-dependent regulation of lysosomes. *Genes Dev.* 30 535–552. 10.1101/gad.274142.115 26944679 PMC4782048

[B157] Zhang J. Wang J. Zhou Z. Park J. E. Wang L. Wu S. (2018). Importance of TFEB acetylation in control of its transcriptional activity and lysosomal function in response to histone deacetylase inhibitors. *Autophagy* 14 1043–1059. 10.1080/15548627.2018.1447290 30059277 PMC6103407

[B158] Zhang T. Zhou Q. Ogmundsdottir M. H. Moller K. Siddaway R. Larue L. (2015). Mitf is a master regulator of the v-ATPase, forming a control module for cellular homeostasis with v-ATPase and TORC1. *J. Cell Sci.* 128 2938–2950. 10.1242/jcs.173807 26092939 PMC4540953

[B159] Zhang X. Cheng X. Yu L. Yang J. Calvo R. Patnaik S. (2016). MCOLN1 is a ROS sensor in lysosomes that regulates autophagy. *Nat. Commun.* 7:12109. 10.1038/ncomms12109 PMC4931332 27357649

[B160] Zhang Z. Qian Q. Li M. Shao F. Ding W. X. Lira V. A. (2020). The unfolded protein response regulates hepatic autophagy by sXBP1-mediated activation of TFEB. *Autophagy* 10.1080/15548627.2020.1788889 Online ahead of print 32597296 PMC8386593

[B161] Zhivotovsky B. Orrenius S. (2010). Cell cycle and cell death in disease: past, present and future. *J. Intern. Med.* 268 395–409. 10.1111/j.1365-2796.2010.02282.x 20964732

