# Supplementary material for: MiT/TFE Family of Transcription Factors: An Evolutionary Perspective
Source: Front Cell Dev Biol. 2021 Jan 6;8:609683. doi: 10.3389/fcell.2020.609683 (PMC7815692; doi:10.3389/fcell.2020.609683)
Supplement: Supplementary file 1 [file Data_Sheet_1.PDF]

Supplementary Table 1: NCBI accession number and UniProt-UniParc ID of the different MiT/TFE proteins analyzed in Fig. 1

Protein name	Accession number	UniProt/UniParc
Hs TFEB	NP_001258873.1	P19484
Mm TFEB	NP_001155195.1	Q9R210
Gg Tfeb	NP_001026093.1	Q5ZM43
Xt Tfeb	NP_001072648.1	Q08D59
Dr Tfeb L	NP_001244121.1	G5CIM3
Dr Tfeb S	XP_005172943.1	UPI000383593B
Hs TFE3	NP_006512.2	P19532
Mm TFE3	NP_766060.2	Q64092
Pc Tfe3	XP_031467342.1	UPI00129DFBD3
Sh Tfe3	XP_030330138.1	UPI0011CFA89D
Dr Tfe3a L	NP_571923.2	Q561Z2
Dr Tfe3a S	XP_005166762.1	UPI00038379F2
Dr Tfe3b	XP_005169362.1	F1R1N1
Hs TFEC	NP_036384.1	O14948
Mm TFEC	XP_006505098.1	Q9WTW4
Gg Tfec	NP_001006229.2	Q5XFQ6
Xt Tfec	XP_002935013.2	A0A5G3L0Y2
Dr Tfec P1	NP_001025276.2	Q1JQ62
Dr Tfec P2	XP_005164592.1	UPI0003837C91
Dr Tfec P3	XM_009299965.1	UPI0000545E20
Hs MITF iso1	NP_937802.1	O75030-1
Hs MITF iso4	NP_000239.1	O75030-9
Mm Mitf isoX2	XP_006505748.1	A0A5H1ZRM6
Mm Mitf isoX10	XP_006505758.1	Q08874
Gg Mitf isoX1	XP_015148452.2	UPI000D63E60E
Gg Mitf isoX8	XP_015148459.1	B6E281
Xt mitf	NP_001093747.1	A4IID0
Dr Mitfa	ENSDART00000056457.5	Q9PWC2
Dr Mitfb	AEP16359.1	F1Q885
Dm Mitfa	NP_001033808.1	Q6WSQ9
Dm Mitfc	NP_001245436.1	C3KGP2
Ce HLH30	NP_500462.1	H2KZZ0
